# Exome Sequencing Identifies a Novel *LMNA* Splice-Site Mutation and Multigenic Heterozygosity of Potential Modifiers in a Family with Sick Sinus Syndrome, Dilated Cardiomyopathy, and Sudden Cardiac Death

**DOI:** 10.1371/journal.pone.0155421

**Published:** 2016-05-16

**Authors:** Michael V. Zaragoza, Lianna Fung, Ember Jensen, Frances Oh, Katherine Cung, Linda A. McCarthy, Christine K. Tran, Van Hoang, Simin A. Hakim, Anna Grosberg

**Affiliations:** 1 UC Irvine Cardiogenomics Program, Department of Pediatrics, Division of Genetics & Genomics and Department of Biological Sciences, University of California Irvine, Irvine, California, United States of America; 2 Department of Biomedical Engineering and The Edwards Lifesciences Center for Advanced Cardiovascular Technology, University of California Irvine, Irvine, California, United States of America; Pennsylvania State University, UNITED STATES

## Abstract

The goals are to understand the primary genetic mechanisms that cause Sick Sinus Syndrome and to identify potential modifiers that may result in intrafamilial variability within a multigenerational family. The proband is a 63-year-old male with a family history of individuals (>10) with sinus node dysfunction, ventricular arrhythmia, cardiomyopathy, heart failure, and sudden death. We used exome sequencing of a single individual to identify a novel *LMNA* mutation and demonstrated the importance of Sanger validation and family studies when evaluating candidates. After initial single-gene studies were negative, we conducted exome sequencing for the proband which produced 9 gigabases of sequencing data. Bioinformatics analysis showed 94% of the reads mapped to the reference and identified 128,563 unique variants with 108,795 (85%) located in 16,319 genes of 19,056 target genes. We discovered multiple variants in known arrhythmia, cardiomyopathy, or ion channel associated genes that may serve as potential modifiers in disease expression. To identify candidate mutations, we focused on ~2,000 variants located in 237 genes of 283 known arrhythmia, cardiomyopathy, or ion channel associated genes. We filtered the candidates to 41 variants in 33 genes using zygosity, protein impact, database searches, and clinical association. Only 21 of 41 (51%) variants were validated by Sanger sequencing. We selected nine confirmed variants with minor allele frequencies <1% for family studies. The results identified *LMNA* c.357-2A>G, a novel heterozygous splice-site mutation as the primary mutation with rare or novel variants in *HCN4*, *MYBPC3*, *PKP4*, *TMPO*, *TTN*, *DMPK* and *KCNJ10* as potential modifiers and a mechanism consistent with haploinsufficiency.

## Introduction

Sick Sinus Syndrome (SSS) is a group of conditions caused by the abnormal pacing of electrical activity via the sinus node; the defect results in atrial bradyarrhythmias (sinus bradycardia and sinus arrest), atrial tachyarrhythmias (atrial fibrillation and atrial flutter), and alternating bradycardia and tachycardia (bradycardia-tachycardia syndrome) [1,2]. Patients may present with fatigue, syncope, and palpitations or may be asymptomatic. Complications include an increased risk for embolic stroke, cardiac dysfunction, and sudden death [1,2].

SSS primarily affects the elderly with an average onset age of 68; however, early onset and familial forms have been documented [1,3]. Inherited forms of SSS are associated with DNA mutations in voltage-gated ion channel genes including *SCN5A* and *HCN4* [4,5]. Compound heterozygous mutations in *SCN5A* were found in three unrelated individuals with congenital SSS; functional studies demonstrated significant defects in sodium channel gating [4]. A heterozygous mutation in *HCN4* was found in a family with sinus bradycardia; functional studies found decreased activity of pacemaker channels [5]. Despite this knowledge, specific mechanisms in how these genetic defects result in heart dysfunction, clinical variability, and disease progression remain unknown.

In this report, we describe a multigenerational family with adult-onset Sick Sinus Syndrome. The goals of this study were first to identify the primary mutation and then to find potential secondary factors that may result in the clinical variation that includes not only sinus node dysfunction but also dilated cardiomyopathy (DCM), ventricular tachycardia (VT), sudden death, and heart failure (HF). We used exome sequencing [6] on a single individual to identify a novel *LMNA* splice-site mutation and demonstrated the importance of validation and co-segregation studies to evaluate the set of candidates. We found multigenic heterozygosity of novel or rare variants as potential secondary factors that may serve a role in intrafamilial variability of *LMNA* disease. In addition, fibroblast studies revealed monoallelic expression of the normal allele which is consistent with haploinsufficiency.

## Materials and Methods

### Study family and ethics statement

**Fig 1:**
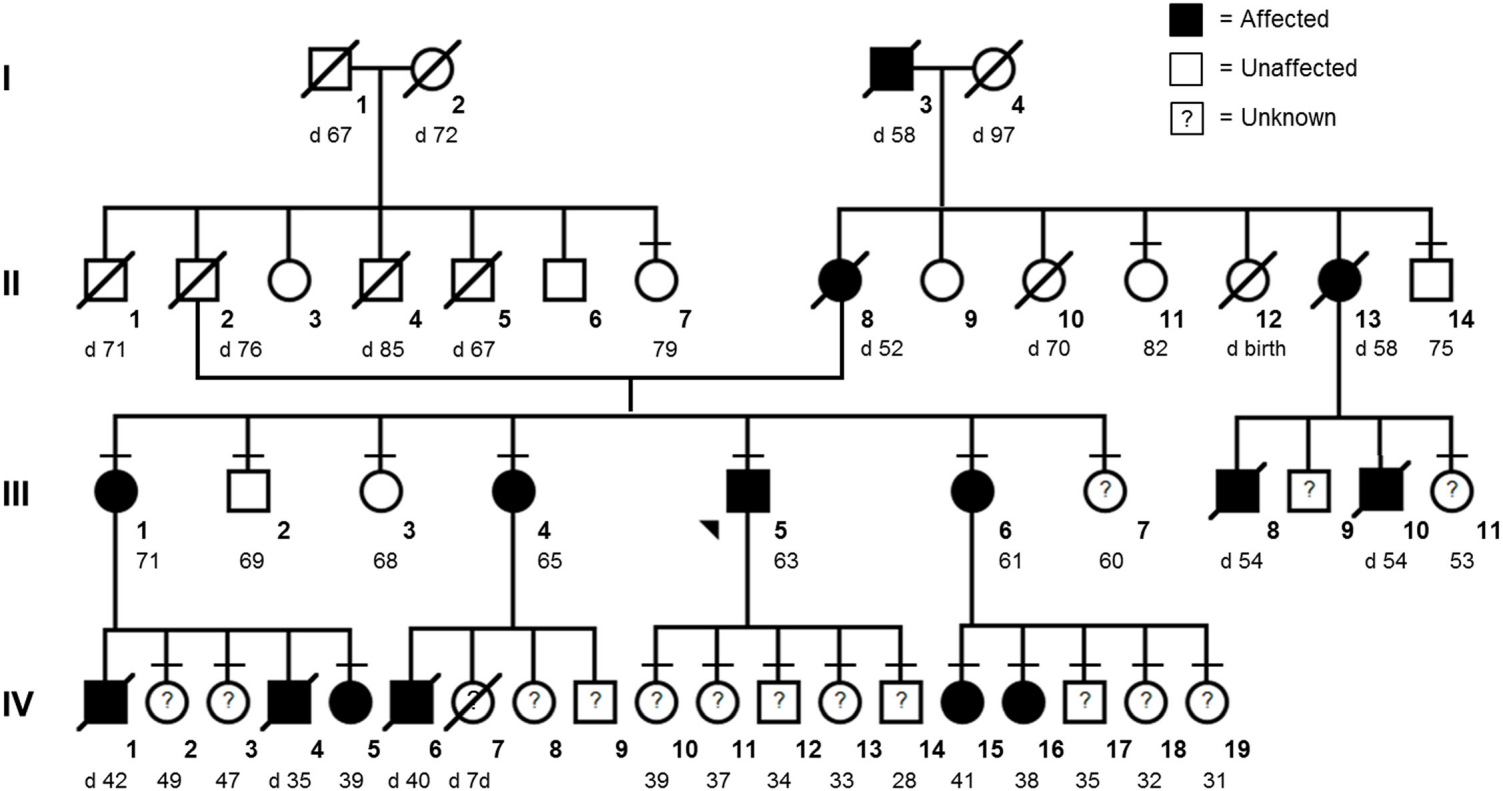
Pedigree. Squares represent males and circles represent females with current age. Short horizontal lines denote individuals consented in the study. Diagonal lines indicate deceased individuals with age of death (d). The proband (III-5) is designated with an arrow. Solid shapes (black) are affected individuals. Unfilled shapes (white) are unaffected individuals defined as those at least 60 years old with no signs/symptoms of cardiac arrhythmia or dysfunction and/or normal cardiac studies. Shapes with a question mark (?) are individuals classified as unknown clinical status. Individual IV-7 died as a neonate; details are not known.

We studied a multigenerational family with Sick Sinus Syndrome (Fig 1). The family has Scottish, German, and Manx ancestry. Informed written consent of all 24 participants (proband and 23 family members) was obtained in accordance with the UC Irvine Institutional Review Board (2007–5577 and 2011–8030) that specifically approved this study. A pedigree, completed questionnaire on health history, medical records, and biological samples were collected for each individual and available medical records reviewed for four deceased individuals (III-8, IV-1, IV-4, IV-6).

Each individual was classified by clinical status as affected, unaffected, or unknown. Affected individuals required documented: 1. arrhythmia including features of SSS (sinus bradycardia, sinus arrest, atrial flutter, atrial fibrillation, or bradycardia-tachycardia syndrome) by standard electrocardiogram (EKG), Holter monitoring, or electrophysiological studies [1,2]; and/or 2. cardiac dysfunction (DCM or HF) without other causes. The diagnosis of DCM was based on systolic dysfunction (ejection fraction (EF) < 45% and/or fractional shortening (FS) <25%) and left ventricular dilation (increased left ventricular end-diastolic diameter (LVEDD)) by echocardiography, radionuclide scanning, or angiography [7]. Heart failure was defined as EF of <40%. Since the age of presentation varied (late 20s to early 50s) in the affected family members (Table 1), we classified an individual as unaffected if the person was at least 60 years old with no signs or symptoms of cardiac disease and/or documentation of normal EKG and/or echocardiography studies. The remaining family members were classified as unknown clinical status.

**Table 1:** Clinical features of the proband and 14 affected family members.

ID	Relationship	Sex	Current age (y)	Px age (y)	Cardiac history (age at diagnosis)
1. I-3^a^	Mat GF	M	d.58	40s	Cardiomyopathy
2. II-8^a^	mother	M	d.52	40s	Arrhythmia & cardiomyopathy; pacemaker and died from CVA(52)
3. II-13^a^	Mat aunt	F	d.58	40s	Arrhythmia & cardiomyopathy
4. III-1	sister	F	71	49	Bradycardia & AF(49); atrial flutter, sinus arrest & pacemaker(52); DCM & HF(61)
5. III-4	sister	F	65	49	Atrial flutter, pacemaker & NSVTx1(49); AF(50); DCM(55); NSVTx1(62)
6. III-5	proband	M	63	54	Atrial flutter(54), VT & ICD(57); CVA(58) & DCM(58); HF(59)
7. III-6	sister	F	61	46	AF, SVT, bradycardia & sinus arrest(46); pacemaker & atrial flutter(49); NSVTx1(58&59)
8. III-8	first cousin	M	d.54	41	Bradycardia(43); AF(44); atrial flutter(45); SSS & NSVT(47) pacemaker(51); DCM, HF, VF & SCD(54)
9. III-10^a^	first cousin	M	d.54	54	Bradycardia, pacemaker, SCD(54)
10. IV-1	nephew	M	d.42	38	DCM, HF & PVCs(38); bradycardia & SVT(39); VT & ICD(40); SCD(42)
11. IV-4	nephew	M	d.35	35	Acute CVA, AF, cardiomyopathy & SCD(35)
12. IV-5	niece	F	39	36	Bradycardia, PVCs, NSVT, mild cardiomyopathy and pacemaker(36)
13. IV-6	nephew	M	d.40	28	DCM, HF, VT & ICD(28); end-stage HF, LVAD & heart transplant(33)
14. IV-15^a^	niece	F	41	32	Sick sinus syndrome(35); pacemaker(38)
15. IV-16	niece	F	38	34	Atrial flutter & AF(34); bradycardia and possible CVA(35)

^a^Information obtained by family history only; medical records were not available for review.

Abbreviations: Px, Presentation; y, years old; Mat, Maternal; GF, grandfather; d., died; CVA, cerebrovascular accident; AF, atrial fibrillation; DCM, dilated cardiomyopathy; HF, heart failure; NSVT, nonsustained ventricular tachycardia; SCD, sudden cardiac death; VF, ventricular fibrillation; VT, ventricular tachycardia; ICD implantable cardiac defibrillator; SVT, supraventricular tachycardia; PVC, premature ventricular complex; LVAD, left ventricular assist device.

Proband: The proband (III-5, Fig 1) is a 63-year-old male who was healthy until age 54 when his EKG showed atrial flutter during evaluation for non-cardiac surgery. Subsequent evaluations led to the diagnosis of SSS. At 54 years old, echocardiogram showed mild systolic dysfunction and left ventricle dilation (EF 45%, LVEDD 58 mm). Myocardial perfusion study showed exercise intolerance but no signs of ischemia. At presentation, he felt well noting mild exercise intolerance for the past year with no palpitations or chest pain; he took no medications. At age 49, he had normal systolic function and LV dilation by echocardiogram (EF 53%, LVEDD 61 mm) and a normal myocardial perfusion study. He had a normal EKG at age 53. Past medical history included nephrectomy and splenectomy after trauma as a child. He has hyperlipidemia. He denied the use of alcohol, tobacco, or drugs. He had a normal birth and development with no signs or symptoms of neuromuscular disease. Serum creatine phosphokinase (CK) levels were normal (CK = 192, 128, and 118 IU/L; normal: 60–400 IU/L).

Affected family members: There are 14 affected family members (Fig 1): six living (three sisters III-1, III-4, III-6 and three nieces IV-5, IV-15, IV-16) and eight deceased individuals (I-3, II-8, II-13, III-8, III-10, IV-1, IV-4, IV-6). The main clinical features are provided in Table 1.

### Molecular studies

Sanger sequencing: To identify the primary mutation in the proband, we conducted DNA sequencing for the 27 exons of *SCN5A* and seven exons of *RYR2* (exons 8, 15, 44, 47, 49, 90, 94). PCR primers sequences were obtain from literature [8] or designed using Primer3. The sequencing data was compared to reference genes using DNA Sequencher (Gene Codes, Ann Arbor, MI). To identify potential mutations from polymorphisms, all DNA variations were analyzed using existing databases including NCBI dbSNP, UCSC Genome Browser, NHLBI ESP Exome Variant Server, and OMIM.

Exome sequencing: DNA was extracted from the proband’s blood using the Gentra Puregene protocol (QIAGEN, Valencia, CA). DNA was sent to Otogenetics (Norcross, GA) for enrichment using the NimbleGen SeqCap EZ Human Exome Library v2.0 (Roche NimbleGen, Madison, WI) and Illumina HiSeq2000 paired-end sequencing (Illumina, San Diego, CA). NimbleGen enrichment targeted 19,056 genes consisting of 194,954 exons; design and annotation files are available online (www.nimblegen.com).

Bioinformatic analysis: The DNAnexus system (DNAnexus, Mountain View, CA) was used to align the data to the human genome reference (hg19). The number of targeted exons sequenced and average read coverage for each exon was determined with DNAnexus Exome Analysis. DNAnexus Nucleotide Level Variation Analysis was utilized to identify DNA variants: single-nucleotide, multi-nucleotide, small insertions, and small deletions and to determine potential zygosity of each variant. A potential heterozygous variant was defined as a nucleotide difference in at least two mapped reads and a potential homozygous variant as a nucleotide difference in all mapped reads.

**Fig 2:**
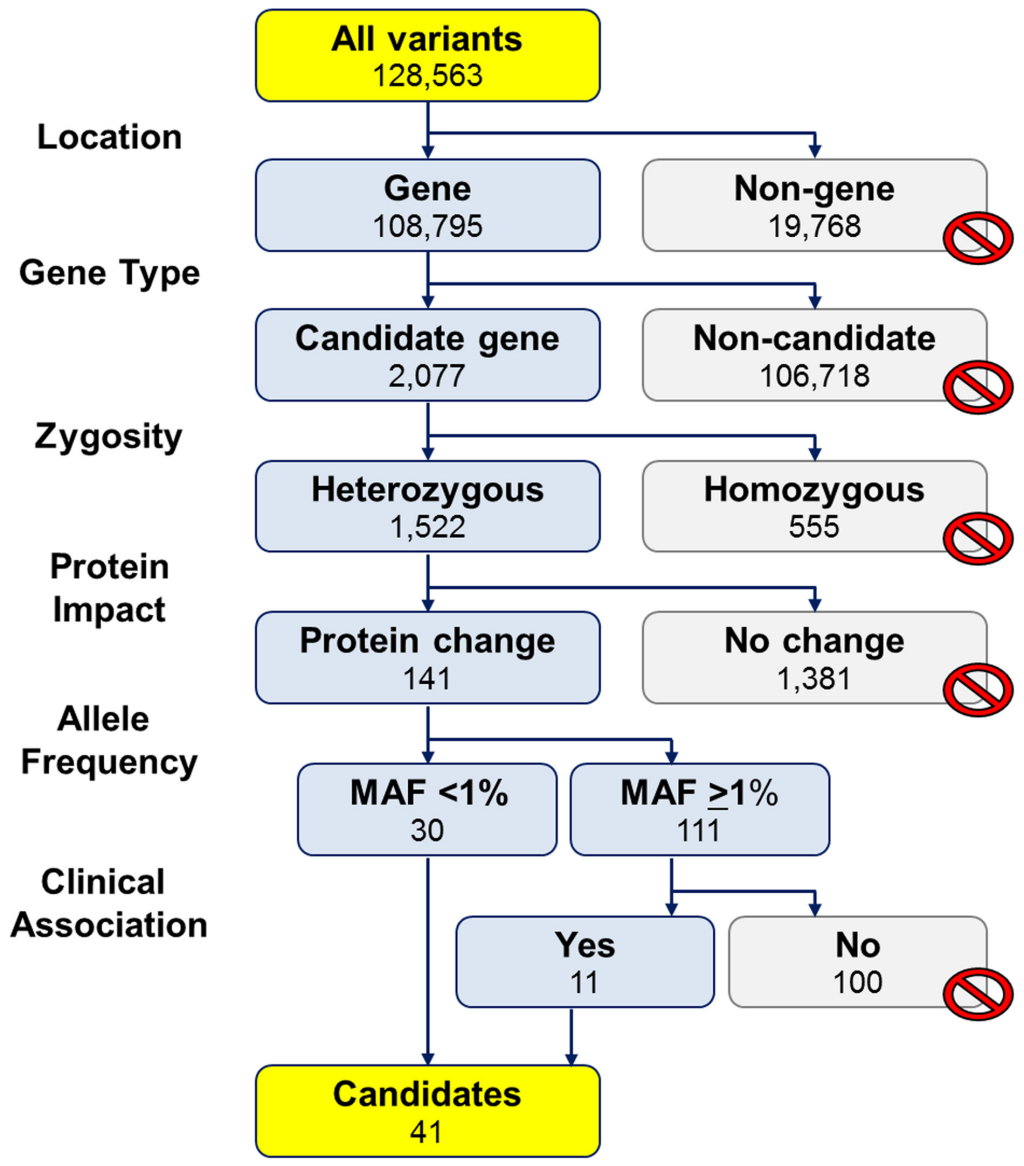
Step-wise approach to identify candidate mutations and potential modifiers. We filtered the 128,563 variants identified from exome sequencing to 41 candidates using six criteria: location (gene or non-gene), gene type (283 arrhythmia, cardiomyopathy or ion channel associated genes), zygosity, predicted protein impact, allele frequency, and clinical association. Types of variants filtered out are marked with a circle with a diagonal line. Abbreviations: MAF, minor allele frequency.

Variant filtering: The list of variants was filtered in a step-wise approach (Fig 2) to identify potential mutations and possible modifiers from benign polymorphisms. First, we filtered by location, either as a variant found within or outside of a protein-coding gene (gene or non-gene). Second, we filtered by gene type focusing on variants found in a list of 283 candidate genes (Appendix A in S1 File) that included 158 genes associated with arrhythmia or cardiomyopathy and 125 genes that encoded for ion channels. These variants were filtered by potential zygosity (heterozygous or homozygous) and then by predicted protein impact (change or no change). Variants that were predicted to alter the protein included non-synonymous amino acid changes (missense), truncation of the polypeptide (nonsense), or potentially altered RNA splicing (splice-site). Splice-site variants were classified as those located in the splice consensus sequences (C/A)AGgt(a/g)agt and cagG for donor and acceptor sites, respectively [9]. Variants that were predicted to result in no change to the encoded protein included those found in an intron, 5’ or 3’ untranslated regions (UTR), and upstream or downstream of the gene. Each variant was classified by allele frequencies. We defined “novel” variants as those absent from databases as described for Sanger sequencing. For known variants, population allele frequencies from NCBI dbSNP (build 135) and NHLBI ESP Exome Variant Server (ESP5400) were used to identify rare variants (minor allele frequencies (MAF) <1%) from low-frequency/common variants (MAF ≥1%). Finally, we used OMIM, Pubmed, and Google searches to find reported clinical associations for known variants.

Validation and family studies: The candidate mutations were validated using Sanger sequencing as described above. The “confirmed variants” were defined as those validated by Sanger sequencing in at least two experiments. To identify the primary mutation, family studies were conducted by Sanger sequencing to determine co-segregation of the confirmed variants and the condition. The presence (+) or absence (-) of the variant was detected by using saliva or blood DNA for nine individuals: four affected (III-1, III-4, III-5, III-6) and five unaffected (II-7, II-11, II-14, III-2, III-3) individuals.

Fibroblast collection and *LMNA* genomic sequencing: After informed consent of the family members (Patients: III-1, III-5, and IV-5 and Family Controls: III-2 and III-3) two dermal punch biopsies were obtained and primary cultures were established using standard procedures. In addition, human dermal fibroblast from two healthy individuals (Non-family Controls) (Lonza Group Ltd, Basel, Switzerland: catalog #CC-2511) were used as unrelated controls. All fibroblasts were cultured in MEM media containing 10% Fetal Bovine Serum (Sigma Aldrich, St. Louis, MO) and 1X HyClone Antibiotic Antimycotic Solution (Thermo Scientific, Waltham, MA). Fibroblasts were harvested at approximately 80–100% confluency for DNA and RNA studies.

Genomic DNA was isolated from fibroblasts using the Blood and Cell Culture DNA Mini Kit (Qiagen, Valencia, CA). Sanger sequencing was conducted for the 12 exons of *LMNA* to confirm the presence of a single mutation in patient fibroblasts and the absence in control fibroblasts and to evaluate for exonic variants. PCR primer sequences were obtained from literature [10] or designed using Primer3 [11] (Table A in S1 File).

Qualitative RNA Analysis of Fibroblasts: To evaluate the impact of the *LMNA* splice site mutation, total RNA was isolated from fibroblasts using the QIAshredder and RNeasy Mini Kit (Qiagen, Valencia, CA) and quantified with a NanoDrop 1000 Spectrophotometer (Thermo Scientific, Waltham, MA). cDNA was synthesized using 100 ng of RNA with the QuantiTect Reverse Transcription Kit (Qiagen, Valencia, CA). Targeted sequences between *LMNA* exons 1–4, 1–10, and 10–11 were PCR amplified from the cDNA with cDNA specific primers designed using NCBI Primer-BLAST [12] (Table A in S1 File). PCR products were resolved using a 2% Gene Pure LE Agarose (ISC BioExpress, Kaysville, UT) gel containing ethidium bromide and imaged with a Universal Hood II Gel Doc (Bio-Rad Laboratories, Irvine, CA).

## Results

The primary goal of this work was to investigate the genetic mechanisms that can contribute to SSS. This work was previously presented in abstract form at the ASHG 2012 meeting: http://www.ashg.org/2012meeting/abstracts/fulltext/f120122793.htm.

### Sanger sequencing: no *SCN5A* and *RYR2* mutations

To accomplish this, we first performed Sanger sequencing and found four previously reported exonic variants in *SCN5A (*rs6599230, rs1805124, rs7430407) and *RYR2 (*rs3765097). Of these, the *SCN5A* rs1805124 (p.His558Arg) was the only non-synonymous variant. *SCN5A* rs1805124 was also found by exome sequencing and was analyzed further as a potential modifier (Table 2).

**Table 2:** Confirmed variants (n = 21) in 18 of 283 genes-known arrhythmia, cardiomyopathy or ion channel genes.

					MAF (%)^a^		
Gene	Position (hg19)	Variant	Predicted impact	dbSNP ID	NCBI	EVS	Clinical association^b^
1. *ACADS*	chr12:121176083	c.625G>A	Gly209Ser; splicing	rs1799958	18	26	Risk allele for SCAD deficiency
2. *ALMS1*^c^	chr2:73675231	c.1579_1580insCTC	Leu527ProLeu	rs34628045	nd	nd	-
3. *CACNA1S*	chr1:201044748	c.1828-5T>C	Splicing	rs1998721	25	22	-
4. *DMPK*^c^	chr19:46274624	c.1616C>T	Thr544Met	rs146680240	0.9	1.3	-
5. *FGA*	chr4:155507590	c.991A>G	Thr331Ala	rs6050	33	25	Risk allele for thromboembolism in AF
6. *HCN4*^c^	chr15:73615097	c.3337A>G	Met1113Val	rs142735148	nd	1.3	Mutation or risk allele for bradycardia
7. *HFE*	chr6:26091336	c.340+4T>C	Splicing	rs2071303	46	33	-
8. *KCNJ10*^c^	chr1:160012270	c.53G>A	Arg18Gln	rs115466046	0.5	1.6	Mutation or risk allele for autism
9. *KCNJ12*	chr17:21319654	c.997_999delGAG	Glu333delGlu	rs142879302	nd	nd	-
10. *KCNK15*	chr20:43378770	c.284A>G	Glu95Gly; splicing	rs1111032	40	50	-
11. *KCNMB1*	chr5:169810796	c.193G>A	Glu65Lys	rs11739136	9.8	10	Risk allele for hypertension (protective)
12. *LMNA*^c^	chr1:156100406	c.357-2A>G	Splicing	rs113610699	nd	nd	-
13. *LMNA*	chr1:156107534	c.1698C>T	His566His; splicing	rs4641	20	25	-
14. *MYBPC3*^c^	chr11:47357479	c.2686G>A	Val895Met	rs35078470	0.3	0.5	Mutation or modifier for HCM
15. *MYH7*	chr14:23900794	c.732C>T	Phe244Phe; splicing	rs2069542	23	18	-
16. *PKP4*^c^	chr2:159519581	c.2384G>A	Arg795Lys	rs139221917	0.1	0.1	-
17. *SCN5A*	chr3:38645420	c.1673A>G	His558Arg	rs1805124	21	23	Risk allele or modifier of arrhythmia
18. *SCN5A*	chr3:38647642	c.1141-3C>A	Splicing	rs41312433	15	19	Risk allele for longer QT interval
19. *TMPO*^c^	chr12:98928103	c.2068C>T	Arg690Cys	rs17028450	0.1	0.1	Mutation for DCM
20. *TTN*^c^	chr2:179587949	c.18053G>C	Thr6018Ser	novel	nd	nd	-
21. *TTN*	chr2:179582537	c.21332C>A	Ala7111Glu; splicing	rs2627043	39	20	-

^a^Population allele frequencies obtained from NCBI dbSNP (build 135) and NHLBI ESP Exome Variant Server (ESP5400, EA cohort only)

^b^Determined by OMIM, Pubmed, and Google searches to find reported clinical associations for known variants.

^c^Variants selected for family studies(n = 9).

Abbreviations: MAF, Minor Allele Frequency; SCAD, Short-chain acyl-CoA dehydrogenase; IVS, intervening sequence; SS, splice site; nd, no data; AF, atrial fibrillation; HCM, hypertrophic cardiomyopathy; DCM, dilated cardiomyopathy.

### Exome sequencing and variant filtering: 41 candidates

To expedite mutation discovery and find potential modifiers, we conducted exome sequencing instead of additional single-gene studies. Based on the pedigree (Fig 1), we hypothesized that the primary mutation was a heterozygous, autosomal dominant variant found in the proband and tracked with all affected family members. We then considered potential mutations that did not track completely with the disease as potential modifiers.

We obtained 9,051,019,760 bases of raw data from 97 million reads. DNAnexus Exome Analysis showed that 94% of reads mapped to hg19 and 98% (191,866 of 194,954) of target exons were sequenced completely. Average mean read coverage of each exon was 129X ± 109X (range: 0 to 7108X), and 92% of the exons had mean coverage above 30X. For the 283 candidate genes, the average mean read coverage of each exon was 109X ± 46X (range: 0 to 1990X) (Table B in S1 File).

We identified 128,563 unique variants with 108,795 (85%) variants located in 16,319 genes of 19,056 target genes (Fig 2). We found 2,077 (1.6%) variants located in 237 of 283 genes in our panel of arrhythmia, cardiomyopathy, and ion channel associated genes. Of these 2,077 variants, we found 1,522 heterozygous variants including 296 coding and 1,258 non-coding variants located in 211 genes. Two variants were in a region that was coding and non-coding in two different isoforms. Of the 1,522 heterozygous variants, we found 141 variants that were predicted to impact the protein. Of these 141 variants, 21 were novel and 120 were known variants. We then filtered the candidates to 41 variants in 33 genes consisting of the 21 novel variants and an additional 20 known variants that were rare (MAF <1%) and/or had a previously reported clinical association.

### Validation and family studies

**Fig 3:**
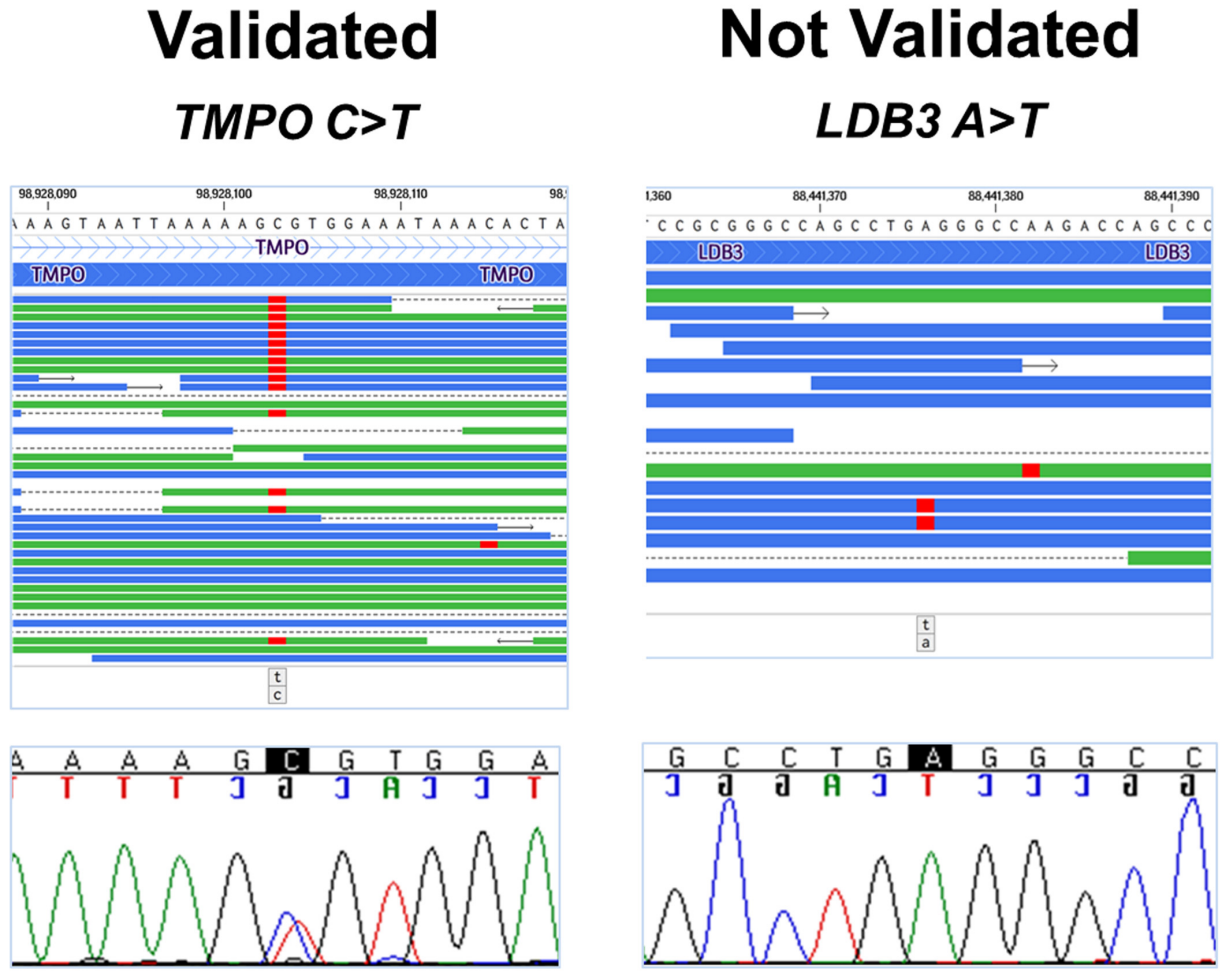
Sanger validation of candidates. Of the filtered 41 candidates, 21 variants were confirmed by Sanger sequencing. Two examples are provided: *TMPO* p.Arg690Cys variant (C>T) for a confirmed variant and an *LDB* variant (A>T) for an unconfirmed variant (artifact). Exome sequencing reads (top) are represented as horizontal lines (forward reads in blue and reverse reads in green); variant nucleotides are shown in red. Sanger sequencing chromatograms (bottom) are provided.

**Fig 4:**
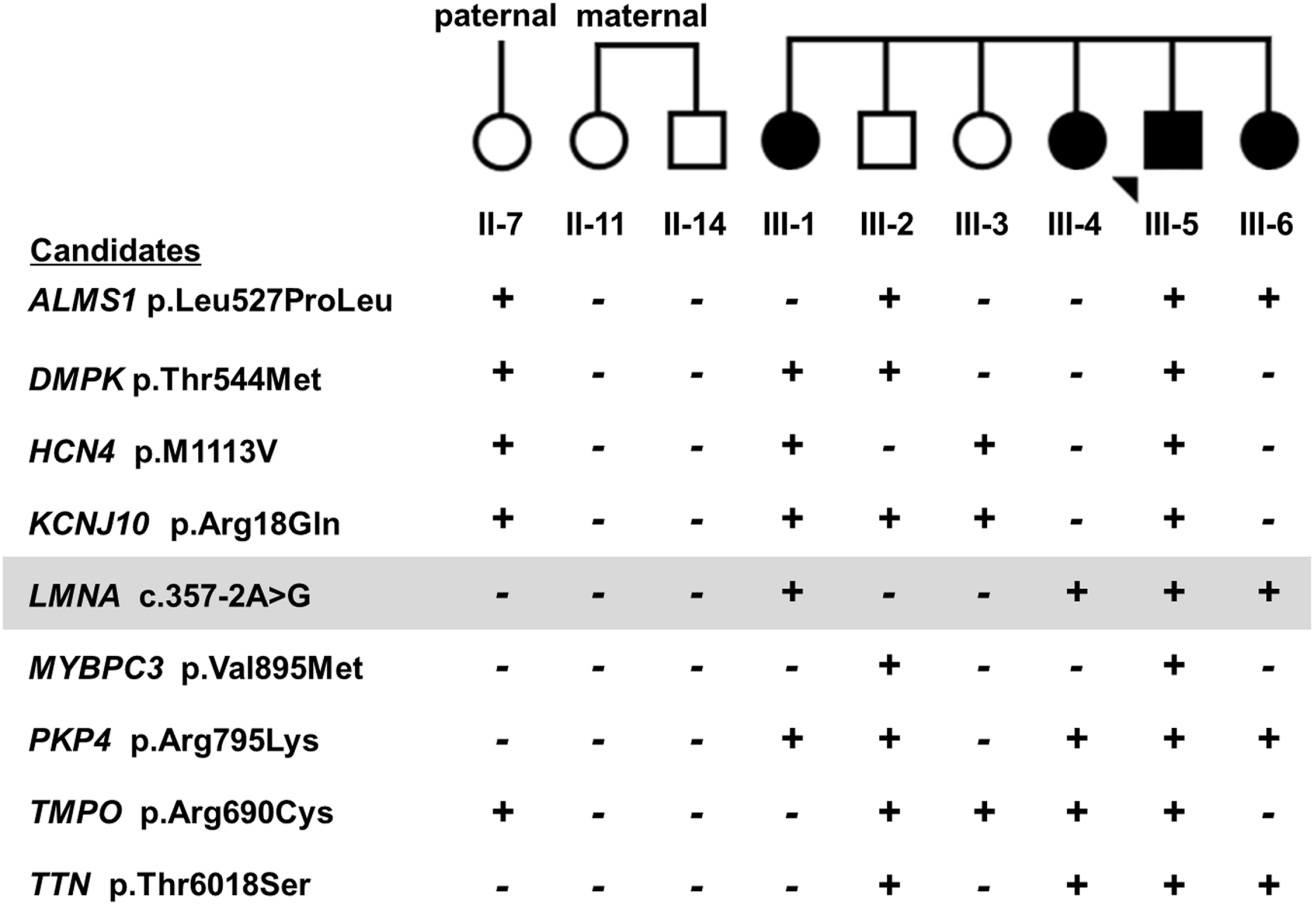
Family studies. We conducted Sanger sequencing for nine family members (top row) to follow the segregation of nine confirmed candidates (first column). The studies included four affected individuals: proband and three sisters and five unaffected individuals: paternal aunt, maternal aunt, maternal uncle, and two siblings. Results depicted as the presence (+) or absence (-) of the DNA variant. Only *LMNA* c.357-2A>G (marked in gray) was present in all affected and absent in all unaffected individuals.

**Fig 5:**
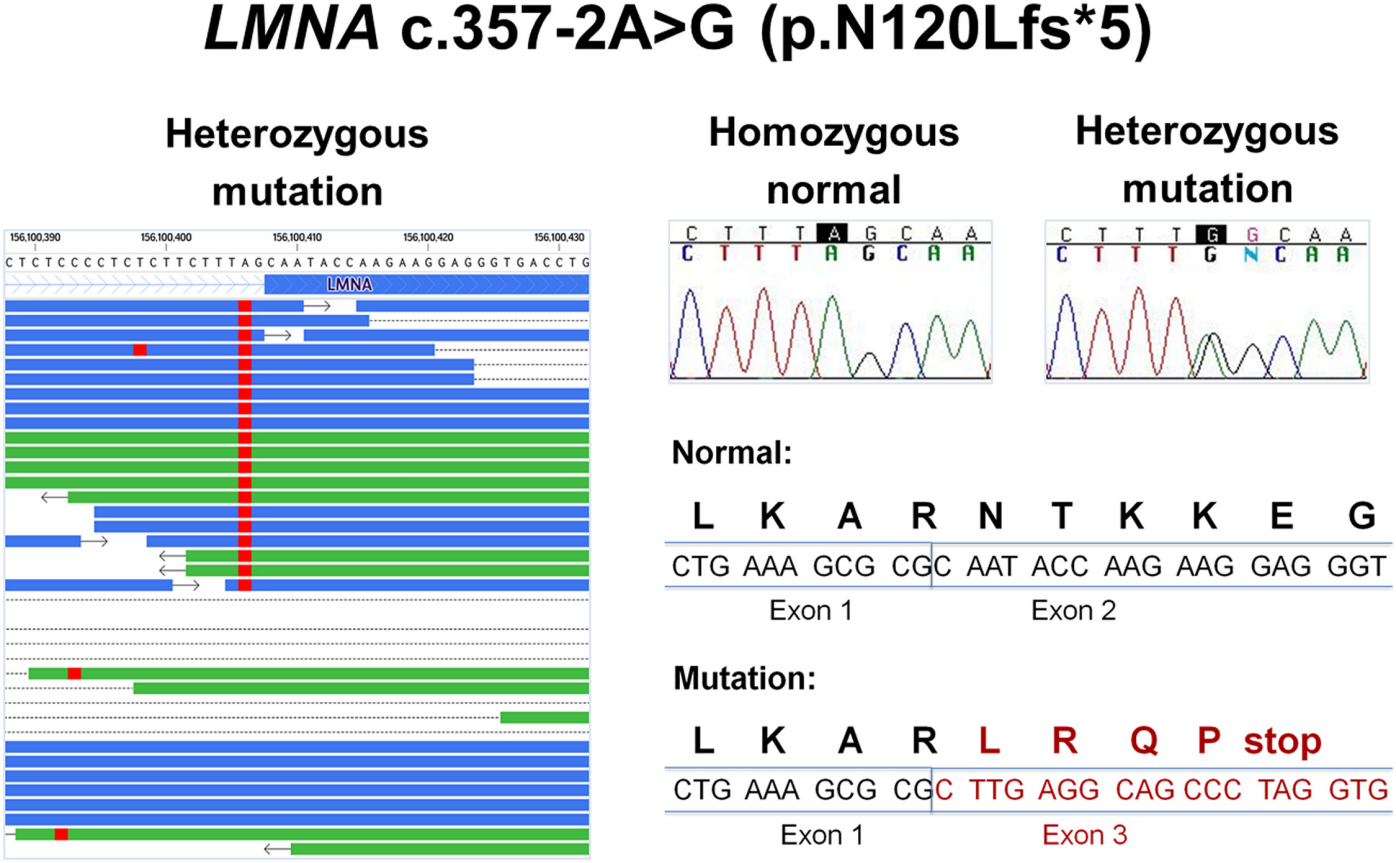
Novel *LMNA* mutation c.357-2A>G (p.N120Lfs*5). Left panel shows the exome sequencing reads of the proband represented as horizontal lines (forward reads in blue and reverse reads in green). Variant nucleotides are shown in red. Right top panel shows the Sanger sequencing chromatograms: normal allele c.357-2A for unaffected individuals and heterozygous mutation c.357-2A>G / c.357-2A for the proband and affected family members. The right bottom panel shows the predicted mRNA and amino acid sequences for the normal and mutated sequences. The mutation is predicted to result in skipping of exon 2 and premature truncation.

We confirmed 21 of 41 (51%) candidates by Sanger sequencing (Table 2 and Fig 3). Compared to known variants, the majority of the novel variants were exome sequencing artifacts. All 20 (100%) known variants were validated compared to only one of 21 (5%) novel variants. The *KCNJ12* variant (c.997_999delGAG) was excluded from further analysis since the result may be due to sequencing of *KCNJ12*-like pseudogenes [13]. Next, family studies were conducted for nine confirmed variants with MAF <1% and/or with previously reported clinical association (Table 2 and Fig 4). These results identified *LMNA* c.357-2A>G (p.N120Lfs*5) (NM_170707.3), a novel heterozygous splice-site mutation as the primary mutation that is predicted to form an aberrant mRNA with an out of frame deletion of exon 2 and introduction of a premature termination codon (Fig 5). The aberrant mRNAs transcribed from the mutated allele would be predicted to be eliminated by nonsense-mediated mRNA decay (NMD). In addition, we found rare or novel heterozygous variants in *HCN4*, *MYBPC3*, *PKP4*, *TMPO*, *TTN*, *DMPK*, and *KCNJ10* as potential modifiers (Table 2).

### RNA studies: no aberrant splicing and monoallelic expression

**Fig 6:**
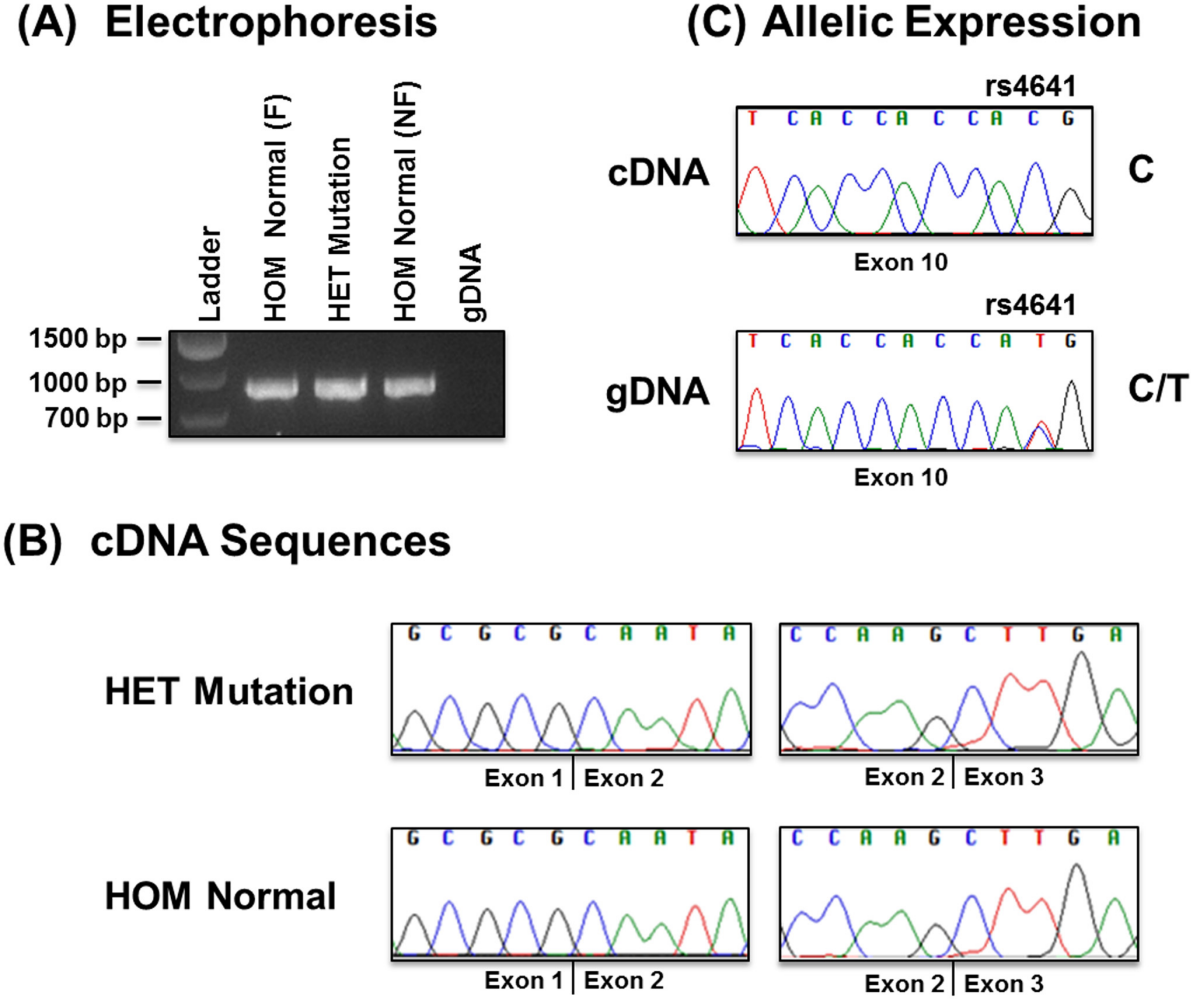
Qualitative Analysis of *LMNA* mRNA in Patient and Control Fibroblasts. (**A**) Electrophoresis: cDNA fragment analysis of Exon 1–4 of *LMNA* isolated from fibroblasts of unaffected age-matched family member III-2 (HOM Normal (F): lane 2), proband with mutation c.357-2A>G (HET Mutation: lane 3), and non-family control (HOM Normal (NF): lane 4). Genomic DNA from a non-family control fibroblast line served as a negative control (lane 5). (**B**) cDNA Sequences: Sequencing of cDNA fragments showing exon junctions between Exon 1–2 and Exon 2–3 for the proband (HET Mutation) and an unaffected age-matched family member (HOM Normal). (**C)** Allelic Expression: Sequences from genomic DNA and cDNA from patient with mutation showing single nucleotide polymorphism rs4641 in exon 10. The top panel shows the monoallelic expression with nucleotide C in the cDNA while the bottom panel shows heterozygosity for C/T in the genomic DNA.

Our qualitative analysis comparing *LMNA* mRNA in fibroblast derived from three patients and four controls (unaffected family members and commercial lines) did not reveal any aberrant splice products (Fig 6a). The sequence analysis of *LMNA* cDNA fragments containing a region between Exon 1–4 indicated that Exon 2 is present in both control and mutant fibroblast lines (Fig 6b). In addition, the sequence analysis of *LMNA* cDNA fragments containing a region between Exon 1–10 did not reveal any other splicing defects.

To determine which *LMNA* allele was expressed, we took advantage of an exonic variant detected in our genomic DNA studies. While these results confirmed the presence of a single heterozygous *LMNA* mutation in all three patient fibroblasts and the absence of *LMNA* protein encoding mutations in all four control fibroblasts, we also found all three patients were heterozygous for a C/T variant located in exon 10 (rs4641: NM_1701707.3:c.1698C>T). These findings are consistent with the *LMNA* splice-site mutation in intron 1 being *in cis* with rs4641 variant T. When we compared *LMNA* genomic and cDNA sequences at exon 10 for the three patients, the cDNA sequence contained C at rs4641 indicating only monoallelic expression of the normal *LMNA* allele (Fig 6c).

## Discussion

### Discovery of the primary mutation by exome sequencing

Due to vast genetic heterogeneity in human cardiomyopathies, traditional “single-gene” approaches are limited when used to identify the primary disease-causing mutation, and new genomic technologies including whole exome sequencing (WES) are now being used [14]. WES uses high-throughput sequencing to catalogue an individual’s DNA variation in over 20,000 protein-encoding genes allowing identification of the primary mutation in patients and families with inherited disease including mitochondrial, dilated, and hypertrophic cardiomyopathies [6,15].

In this work, WES of a single affected individual was applied to find the primary disease-causing mutation in a family with SSS, DCM, and sudden death. Recently, three other studies used WES of multiple individuals to identify *LMNA* mutations in families with similar clinical features. WES of six members (five affected and one unaffected) identified a novel *LMNA* nonsense mutation (p.Arg225X) in a Japanese family [16], and WES of four members (three affected and one unaffected) found a known *LMNA* missense mutation (p.Lys219Thr) in an Italian family [17]. WES of five members (three affected and two unaffected) identified a novel *LMNA* missense mutation (p.Gly232Val) in a Chinese family [18]. In these three studies, WES of multiple family members had several advantages compared to WES of only a single individual. One advantage is the reduction of WES artifacts by focusing on shared variants among affected family members [6].

Our study demonstrated WES of a single affected individual can be done successfully to identify the primary mutation with two critical steps: validation and family studies. Validation was necessary in this study because of the high level of WES false-positive variants. After filtering, 41 WES variants were candidates with only 21 validated by Sanger sequencing. Indeed, nearly all novel candidate variants were WES artifacts; in contrast, all known variants were validated. These false-positive variants have been seen previously with such techniques and may be due to a number of factors in variant calling including read coverage, data quality, enrichment method, sequence alignment, and bioinformatics pipeline [6,19,20]. In retrospect, the number of false-positives might have been reduced if we used increased stringency for data quality, read coverage, and heterozygosity calling [21]. While WES of additional individuals may have reduced the number of artifacts; however, false-positive variants cannot be eliminated completely [18]. Until there is accurate and consistent variant calling for WES, our results demonstrate the value of validation especially for all novel variants identified in a single individual.

The second critical step to identify the primary mutation was family studies to investigate co-segregation and to properly identify the primary mutation among multiple candidates that included reported putative mutations. We used Sanger analysis of candidate variants in multiple affected individuals (three siblings) but also five unaffected individuals (>60 years old). We found only the *LMNA* c.357-2A>G variant completely segregated with the phenotype and was not found in all five unaffected individuals.

In contrast to previous reports, our study does not support the role of *TMPO* p.Arg690Cys [22], *MYBPC3* p.Val895Met [23,24], and *HCN4* p.Met1113Val [25] as highly penetrant, primary mutations. We excluded *TMPO* p.Arg690Cys as the primary mutation in our family although this variant was implicated as a DCM mutation [22]. Since the lamin A protein interacts with Thymopoietin (TMPO), now designated as lamina-associated polypeptide 2 (LAP2), a DCM cohort was screened for *TMPO* mutations. The *TMPO* p.Arg690Cys variant was found in two brothers with severe DCM; functional studies supported pathogenicity that showed reduced lamin A binding of the *TMPO* p.Arg690Cys protein *in vitro* [22]. Our family studies showed that two of three affected siblings and three unaffected family members (79-year-old paternal aunt and two older siblings), had the variant; therefore, we concluded that *TMPO* p.Arg690Cys variant was not the primary mutation in our family. This may suggest that additional factors may contribute to the expression of *TMPO/LAP2*-associated DCM.

Our study also does not support the role of *MYBPC3* p.Val895Met as a highly penetrant, primary mutation. *MYBPC3* mutations have been known to contribute hypertrophic cardiomyopathy (HCM); however, the role of the *MYBPC3* p.Val895Met variant is not clear. As evidence for pathogenicity, pVal895Met was found in a single “white” patient [23] and five patients from two unrelated Chinese families [24]. The variant also was considered a potential modifier of HCM after it was found in a European HCM family with a second variant *MYH7* A355T; one affected individual had only the *MYH7* variant and three affected had both variants including one patient with a severe phenotype [26]. Since only the proband and his 69-year-old unaffected brother had the *MYBPC3* p.Val895Met variant, we concluded that the variant is not the primary cause for disease in our family.

Finally, *HCN4* p.Met1113Val was another interesting candidate since *HCN4* mutations contribute to familial sinus bradycardia [5] with preliminary evidence for an association of *HCN* p.M1113V and post-partum sinus bradycardia [25]. Among 15 affected women; one subject had a previously published *HCN4* mutation and two subjects had the *HCN* p.M1113V variant [25]. In our family, we found the lack of segregation for the *HCN* p.M1113V and heart disease; only one affected sibling but also two unaffected family members had this variant including a healthy 79-year-old paternal aunt.

### Lamin A/C and the predicted impact of *LMNA* c.357-2A>G

Primary mutations in *LMNA* are well-documented causes of inherited diseases collectively called the “laminopathies” that include isolated cardiac disease with arrhythmia and DCM [10,27,28]; skeletal myopathies: Emery-Dreifuss muscular dystrophy and limb-girdle muscular dystrophy; premature aging syndromes as Hutchinson-Gilford progeria syndrome (HGPS); and lipodystrophies. *LMNA* is located at chromosome 1q22, contains 12 exons, and encodes multiple isoforms through differential splicing including lamin A and lamin C. The protein structure for lamin A includes an N-terminal globular head, a long central alpha helical rod domain with four segments (1A, 1B, 2A, and 2B), and a C-terminal tail that contains an immunoglobulin-like fold region, nuclear translocation signal, and CAAX box used for post-translational modifications [29]. The lamin proteins assemble into intermediate filaments and higher order structures to form the nuclear lamina, the structural matrix of the nuclear envelope that interacts with both the cell nucleus and cytoskeleton [30]. Through these interactions, lamin A/C plays key roles in nuclear architecture and stability, cytoskeletal mechanics, and multiple nuclear functions including signal transduction and chromatin regulation [30].

We report *LMNA* c.357-2A>G, a novel transition at the splice acceptor site in the first intron as the primary mutation in a family with SSS and DCM. A mutation at the adjacent nucleotide in the same splice acceptor site, *LMNA* c.357-1G>T was reported in three patients with arrhythmia and DCM [31,32]. In a cohort of 324 DCM patients, *LMNA* c.357-1G>T was detected in one family that consisted of a 64 year-old proband with heart transplantation at age 61, atrial fibrillation, and a pacemaker/ICD and his 33 year-old son diagnosed with heart failure at age 28, premature ventricular contractions (PVCs), and a pacemaker/ICD [31]. *LMNA* c.357-1G>T also was found in one French patient with sporadic DCM, a 34 year-old male with NYHA class III heart failure, LBBB and atrial fibrillation [32].

To investigate the molecular mechanism by which *LMNA* c.357-2A>G leads to adult-onset SSS and DCM in our family, we conducted RNA studies on patient and control fibroblast. Our results are consistent with haploinsufficiency due to monoallelic expression of the normal *LMNA* allele in mutant fibroblast. Although the *LMNA* c.357-2A>G mutation is predicted to result in transcription of aberrant mRNAs with an out of frame deletion of exon 2 and introduction of a premature termination codon (PTC), we found no evidence for aberrant *LMNA* splicing. These results suggest that the aberrant mRNAs transcribed from the mutated allele were eliminated by nonsense-mediated mRNA decay (NMD) as described for other *LMNA* mutations [33–35].

Surprisingly, our findings were consistent with haploinsufficiency due to NDM that conflicted with the RNA studies conducted for the *LMNA* c.357-1G>T family [31]. Since both mutations are at the same splice site, we expected *LMNA* c.357-1G>T and *LMNA* c.357-2A>G to both produce the same aberrant products that would be subject to NMD. However, for the *LMNA* c.357-1G>T, RNA studies showed skipping of exon 2 in the explanted heart of the proband and expression of the aberrant transcript [31]. The lack of elimination by NMD and subsequent translation would result in a truncated mutant lamin A protein (124 amino acids) that might have dominant-negative effects on the structural matrix of the nuclear envelope and cytoskeletal mechanics. Evidence for this mechanism by protein studies was not provided [31].

One possible explanation for these conflicting results may be due to tissue-specific differences: the RNA analysis of *LMNA* c.357-1G>T was performed in heart tissue compared to our studies in fibroblasts. Differences in NMD mRNA decay efficiency have been found between various tissues in a mouse model [36]. Human studies have also shown PTC mutations that result in NMD in one tissue and transcription of aberrant RNAs in a different tissue [37]. This includes RNA studies from explanted heart tissue and cultured fibroblasts from a family with a *LMNA* nonsense mutation [38]. The role of “incomplete NMD” was suggested after a weak reduction of mutant transcripts was observed in heart tissue compared to skin fibroblasts. Thus, it is possible that there are tissue-specific differences in the efficiency of NMD in the heart and fibroblasts that result in different mRNA surveillance outcomes. As heart and muscle tissue are not available from our family, our future studies will include RNA and protein analysis of cardiomyocytes differentiated from patient-derived induced pluripotent stem cells. These studies may provide additional evidence for haploinsufficiency in fibroblast and a dominant negative effect in the heart resulting from tissue-specific differences in NMD efficiency.

### Intrafamilial variability and potential *LMNA* modifiers

The clinical features, phenotypic spectrum, and types of *LMNA* mutations have been well-documented for isolated cardiac disease with arrhythmia and DCM [10,27–29]. Our family displayed the typical features of conduction system disease, DCM, risk for death, and an absence of skeletal muscle disease. Affected individuals also had typical disease course that began in middle age [10] and often progressed with age: atrial enlargement and bradyarrhythmias in the 2nd-3rd decades, AV heart block in the 3rd-4th decades, atrial flutter and atrial fibrillation in the 4th-5th decade, and then left ventricular enlargement, systolic dysfunction, heart failure, ventricular arrhythmias, and thromboembolism [28,39,40].

We also noted considerable intrafamilial variability amongst affected individuals in our family. Intrafamilial variability has been reported within large multigenerational *LMNA* families [39–43]. One of the largest families is a German family with a *LMNA* mutation (c.906_907delCT) consisting of nine generations and nearly 500 family members [39,40,44]. Although the disease progressed in distinct stages, there was considerable variation in pacemaker implantation [39], age of onset [40], and disease severity [43] among affected individuals. In addition, sudden death clustered in the different family lineages; more than half of the individuals with sudden death had a parent who died by the same way [39]. Our family showed considerable intrafamilial variability that involved an earlier age of presentation and increased severity of disease in the generation after the proband (Table 1). The average age of presentation for affected individuals in generation III (33.8 ± 3.5 years old, n = 6) was lower compared to that in generation II (48.8 ± 5.0 years old, n = 6). The earlier disease presentation may be due to early clinical screening in subsequent generations due to the family history. However, sudden death also occurred younger in generation III (39 years old versus 54 years old). Therefore, additional factors need to be considered.

Intrafamilial variability has been attributed to a number of factors: early clinical screening, environmental factors, age, gender, response to therapies, and genetic factors; however, the role of these factors in the relationship between genotype and phenotype is not understood [44–46]. By WES, we identified rare and novel variants in other cardiomyopathy/arrhythmia-associated genes, and we speculate that these may serve as potential genetic modifiers. Genetic modifiers are defined as secondary DNA variants that change the phenotypic outcome of the primary mutation in another gene [47]. The secondary DNA variant(s) may modulate the effects of the primary mutation through altered penetrance, dominance modification, or changing expressivity [47].

Only a few examples of genetic modifiers have been described in humans; thus, their mechanisms are poorly understood [48,49]. This includes a possible “digenic” mechanism to explain variability within a family with the *LMNA* R644C mutation and a second mutation in Desmin (*DES*) [50] and in another family with a *de novo LMNA* S326T mutation and a second mutation in the X-linked Emerin (*EMD*) gene [51]. Individuals with two “single-gene” mutations, one in *LMNA* and a second in *DES* or *EMD* had early-disease onset and increased severity [50,51]. More recently, a family with variability for DCM was discovered to have a *LMNA* K219T mutation and a second mutation in Titin (*TTN*) [17]. Family members with two “single-gene” mutations had an earlier age of heart transplantation compared to those with only the *LMNA* mutation which suggests *TTN* may act as modifier of *LMNA* DCM [17].

By WES studies, we found many potential modifiers that will require further analysis (Table 2) including a novel *TTN* p.Thr6018Ser variant, the rare variants reported as putative mutations including *TMPO* p.Arg690Cys [22] and p.*MYBPC3* Val895Met [23,24,26], and common variants including p.*SCN5A* His558Arg associated with increased risk for arrhythmia [52]. In addition, we found *PKP4* p.Arg795Lys in all four affected members and in one unaffected member (III-2). Digenic heterozygosity of mutations in desmosomal genes such as *PKP4* gene has been found to play a role in the variable phenotype in arrhythmogenic right ventricular cardiomyopathy [53]. Further studies are needed to determine the role of digenic heterozygosity of *LMNA* and each potential modifier gene in the variable phenotype of our family.

We also need to consider that interfamilial variability may involve complex multigenic interactions [44–46]. For each individual in population studies, a vast amount of DNA variation was seen that includes >1 million variants throughout the genome, >20,000 variants in the protein encoding regions that include hundreds of rare nonsynonymous and potentially damaging variants at highly conserved positions [6,54]. With these observations, new models are emerging in which the primary mutation is insufficient, and a combination of variants, an individual’s “unique composition of his or her genome-wide mutation burden,” may serve to modulate age of onset and severity of disease [46,55]. Therefore, a unique composition of variants would be expected using high-throughput sequencing as observed in our study and other studies in patients with arrhythmia and cardiomyopathy [56,57].

## Conclusions

We identified a novel *LMNA* splice-site mutation by exome sequencing in a family with DCM and arrhythmia. The fibroblast studies that were conducted showed monoallelic expression of the normal allele which is consistent with a haploinsufficient mechanism. In addition to that mutation, we also discovered multigenic heterozygosity of novel/rare variants in known arrhythmia, cardiomyopathy, or ion channels associated genes that may serve as potential secondary factors in the intrafamilial variability of the disease. We emphasize that the approach in this present study was used to facilitate the selection of *potential* modifiers. Future studies will focus on the development of functional models to test tissue-specific differences in the NMD efficiency and potential *LMNA* modifiers that may influence age of onset (early versus late-onset) and disease severity between generations. We hope that the results will help us to understand the molecular basis of the clinical variability in *LMNA* cardiomyopathy and arrhythmia.

## Supporting Information

S1 File Supplementary materials. (DOCX)

## References

[1] Adan V, Crown LA (2003) Diagnosis and treatment of sick sinus syndrome. Am Fam Physician 67: 1725–1732. 12725451

[2] Ferrer MI (1973) The sick sinus syndrome. Circulation 47: 635–641. 4571058 10.1161/01.cir.47.3.635

[3] Bacos JM, Eagan JT, Orgain ES (1960) Congenital familial nodal rhythm. Circulation 22: 887–895. 13685714 10.1161/01.cir.22.5.887

[4] Benson DW, Wang DW, Dyment M, Knilans TK, Fish FA, Strieper MJ, et al (2003) Congenital sick sinus syndrome caused by recessive mutations in the cardiac sodium channel gene (SCN5A). J Clin Invest 112: 1019–1028. 14523039 10.1172/JCI18062 PMC198523

[5] Milanesi R, Baruscotti M, Gnecchi-Ruscone T, DiFrancesco D (2006) Familial sinus bradycardia associated with a mutation in the cardiac pacemaker channel. N Engl J Med 354: 151–157. 16407510 10.1056/NEJMoa052475

[6] Bamshad MJ, Ng SB, Bigham AW, Tabor HK, Emond MJ, Nickerson DA, et al (2011) Exome sequencing as a tool for Mendelian disease gene discovery. Nature Reviews Genetics 12: 745–755. 10.1038/nrg3031 21946919

[7] Mestroni L, Maisch B, McKenna WJ, Schwartz K, Charron P, Rocco C, et al (1999) Guidelines for the study of familial dilated cardiomyopathies. Collaborative Research Group of the European Human and Capital Mobility Project on Familial Dilated Cardiomyopathy. Eur Heart J 20: 93–102. 10099905 10.1053/euhj.1998.1145

[8] Priori SG, Napolitano C, Tiso N, Memmi M, Vignati G, Bloise R, et al (2001) Mutations in the cardiac ryanodine receptor gene (hRyR2) underlie catecholaminergic polymorphic ventricular tachycardia. Circulation 103: 196–200. 11208676 10.1161/01.cir.103.2.196

[9] Mount SM (1982) A catalogue of splice junction sequences. Nucleic Acids Res 10: 459–472. 7063411 10.1093/nar/10.2.459 PMC326150

[10] Fatkin D, MacRae C, Sasaki T, Wolff MR, Porcu M, Frenneaux M, et al (1999) Missense mutations in the rod domain of the lamin A/C gene as causes of dilated cardiomyopathy and conduction-system disease. N Engl J Med 341: 1715–1724. 10580070 10.1056/NEJM199912023412302

[11] Untergasser A, Cutcutache I, Koressaar T, Ye J, Faircloth BC, Remm M, et al (2012) Primer3-new capabilities and interfaces. Nucleic Acids Research 40. 10.1093/nar/gks596 PMC3424584 22730293

[12] Ye J, Coulouris G, Zaretskaya I, Cutcutache I, Rozen S, Madden TL (2012) Primer-BLAST: a tool to design target-specific primers for polymerase chain reaction. BMC Bioinformatics 13: 134 10.1186/1471-2105-13-134 22708584 PMC3412702

[13] Postma A, Bhuiyan ZA, Shkolnikova M, Denjoy I, Mannens MM, Wilde AA, et al (2004) Involvement of the Kir2 gene family in catecholaminergic polymorphic ventricular tachycardia; analysis for mutation and identification of numerous pseudogenes. Eur Heart J 25: 66.

[14] Sturm AC, Hershberger RE (2013) Genetic testing in cardiovascular medicine: current landscape and future horizons. Curr Opin Cardiol 28: 317–325. 10.1097/HCO.0b013e32835fb728 23571470

[15] Norton N, Li D, Hershberger RE (2012) Next-generation sequencing to identify genetic causes of cardiomyopathies. Curr Opin Cardiol 27: 214–220. 10.1097/HCO.0b013e328352207e 22421630

[16] Arimura T, Onoue K, Takahashi-Tanaka Y, Ishikawa T, Kuwahara M, Setou M, et al (2013) Nuclear accumulation of androgen receptor in gender difference of dilated cardiomyopathy due to lamin A/C mutations. Cardiovasc Res 99: 382–394. 10.1093/cvr/cvt106 23631840

[17] Roncarati R, Viviani Anselmi C, Krawitz P, Lattanzi G, von Kodolitsch Y, Perrot A, et al (2013) Doubly heterozygous LMNA and TTN mutations revealed by exome sequencing in a severe form of dilated cardiomyopathy. Eur J Hum Genet 21: 1105–1111. 10.1038/ejhg.2013.16 23463027 PMC3778353

[18] Lai CC, Yeh YH, Hsieh WP, Kuo CT, Wang WC, Chu CH, et al (2013) Whole-exome sequencing to identify a novel LMNA gene mutation associated with inherited cardiac conduction disease. PLoS One 8: e83322 10.1371/journal.pone.0083322 24349489 PMC3861486

[19] Fuentes Fajardo KV, Adams D, Mason CE, Sincan M, Tifft C, Toro C, et al (2012) Detecting false-positive signals in exome sequencing. Hum Mutat 33: 609–613. 10.1002/humu.22033 22294350 PMC3302978

[20] O'Rawe J, Jiang T, Sun G, Wu Y, Wang W, Hu J, et al (2013) Low concordance of multiple variant-calling pipelines: practical implications for exome and genome sequencing. Genome Med 5: 28 10.1186/gm432 23537139 PMC3706896

[21] Heinrich V, Stange J, Dickhaus T, Imkeller P, Kruger U, Bauer S, et al (2012) The allele distribution in next-generation sequencing data sets is accurately described as the result of a stochastic branching process. Nucleic Acids Res 40: 2426–2431. 10.1093/nar/gkr1073 22127862 PMC3315291

[22] Taylor MR, Slavov D, Gajewski A, Vlcek S, Ku L, Fain PR, et al (2005) Thymopoietin (lamina-associated polypeptide 2) gene mutation associated with dilated cardiomyopathy. Hum Mutat 26: 566–574. 16247757 10.1002/humu.20250

[23] Moolman-Smook JC, De Lange WJ, Bruwer EC, Brink PA, Corfield VA (1999) The origins of hypertrophic cardiomyopathy-causing mutations in two South African subpopulations: a unique profile of both independent and founder events. Am J Hum Genet 65: 1308–1320. 10521296 10.1086/302623 PMC1288283

[24] Wang AL, Kong DH, Chen DX, Wan J, Yu YX (2010) Mutation of V896M in cardiac myosin binding protein-c gene in two Chinese families with hypertrophic cardiomyopathy. Mol Med Rep 3: 759–763. 10.3892/mmr.2010.333 21472310

[25] Nof E, Miller L, Kuperstein R, Eldar M, Glikson M, Luria D (2011) Acquired Post Patrum Bradycardia: Clinical Features Associated with Mutations in the hyperpolarization-activated nucleotide-gated channel 4 Cardiac Ion Channel. Europace 13: 670.

[26] Richard P, Charron P, Carrier L, Ledeuil C, Cheav T, Pichereau C, et al (2003) Hypertrophic cardiomyopathy: distribution of disease genes, spectrum of mutations, and implications for a molecular diagnosis strategy. Circulation 107: 2227–2232. 12707239 10.1161/01.CIR.0000066323.15244.54

[27] Arbustini E, Pilotto A, Repetto A, Grasso M, Negri A, Diegoli M, et al (2002) Autosomal dominant dilated cardiomyopathy with atrioventricular block: A lamin A/C defect-related disease. Journal of the American College of Cardiology 39: 981–990. 11897440 10.1016/s0735-1097(02)01724-2

[28] Hershberger RE, Morales A (1993) Dilated Cardiomyopathy Overview In: Pagon RA, Adam MP, Ardinger HH, Wallace SE, Amemiya A et al, editors. GeneReviews(R). Seattle (WA). 20301486

[29] Dittmer TA, Misteli T (2011) The lamin protein family. Genome Biol 12: 222 10.1186/gb-2011-12-5-222 21639948 PMC3219962

[30] Dechat T, Adam SA, Taimen P, Shimi T, Goldman RD (2010) Nuclear Lamins. Cold Spring Harbor Perspectives in Biology 2. 10.1101/cshperspect.a000547 PMC2964183 20826548

[31] Parks SB, Kushner JD, Nauman D, Burgess D, Ludwigsen S, Peterson A, et al (2008) Lamin A/C mutation analysis in a cohort of 324 unrelated patients with idiopathic or familial dilated cardiomyopathy. American Heart Journal 156: 161–169. 10.1016/j.ahj.2008.01.026 18585512 PMC2527054

[32] Millat G, Bouvagnet P, Chevalier P, Sebbag L, Dulac A, Dauphin C, et al (2011) Clinical and mutational spectrum in a cohort of 105 unrelated patients with dilated cardiomyopathy. Eur J Med Genet 54: e570–575. 10.1016/j.ejmg.2011.07.005 21846512

[33] Narula N, Favalli V, Tarantino P, Grasso M, Pilotto A, Bellazzi R, et al (2012) Quantitative Expression of the Mutated Lamin A/C Gene in Patients With Cardiolaminopathy. Journal of the American College of Cardiology 60: 1916–1920. 10.1016/j.jacc.2012.05.059 23062543

[34] Muchir A, van Engelen BG, Lammens M, Mislow JM, McNally E, Schwartz K, et al (2003) Nuclear envelope alterations in fibroblasts from LGMD1B patients carrying nonsense Y259X heterozygous or homozygous mutation in lamin A/C gene. Experimental Cell Research 291: 352–362. 14644157 10.1016/j.yexcr.2003.07.002

[35] Al-Saaidi R, Rasmussen TB, Palmfeldt J, Nissen PH, Beqqali A, Hansen J, et al (2013) The LMNA mutation p.Arg321Ter associated with dilated cardiomyopathy leads to reduced expression and a skewed ratio of lamin A and lamin C proteins. Exp Cell Res 319: 3010–3019. 10.1016/j.yexcr.2013.08.024 24001739

[36] Zetoune AB, Fontaniere S, Magnin D, Anczukow O, Buisson M, Zhang CX, et al (2008) Comparison of nonsense-mediated mRNA decay efficiency in various murine tissues. BMC Genet 9: 83 10.1186/1471-2156-9-83 19061508 PMC2607305

[37] Bateman JF, Freddi S, Nattrass G, Savarirayan R (2003) Tissue-specific RNA surveillance? Nonsense-mediated mRNA decay causes collagen X haploinsufficiency in Schmid metaphyseal chondrodysplasia cartilage. Hum Mol Genet 12: 217–225. 12554676 10.1093/hmg/ddg054

[38] Geiger SK, Bar H, Ehlermann P, Walde S, Rutschow D, Zeller R, et al (2008) Incomplete nonsense-mediated decay of mutant lamin A/C mRNA provokes dilated cardiomyopathy and ventricular tachycardia. J Mol Med (Berl) 86: 281–289. 17987279 10.1007/s00109-007-0275-1

[39] Graber HL, Unverferth DV, Baker PB, Ryan JM, Baba N, Wooley CF (1986) Evolution of a hereditary cardiac conduction and muscle disorder: a study involving a family with six generations affected. Circulation 74: 21–35. 3708775 10.1161/01.cir.74.1.21

[40] Sparks EA, Boudoulas KD, Raman SV, Sasaki T, Graber HL, Nelson SD, et al (2011) Heritable cardiac conduction and myocardial disease: from the clinic to the basic science laboratory and back to the clinic. Cardiology 118: 179–186. 10.1159/000328638 21691096

[41] Jakobs PM, Hanson EL, Crispell KA, Toy W, Keegan H, Schilling K, et al (2001) Novel lamin A/C mutations in two families with dilated cardiomyopathy and conduction system disease. J Card Fail 7: 249–256. 11561226 10.1054/jcaf.2001.26339

[42] Hershberger RE, Hanson EL, Jakobs PM, Keegan H, Coates K, Bousman S, et al (2002) A novel lamin A/C mutation in a family with dilated cardiomyopathy, prominent conduction system disease, and need for permanent pacemaker implantation. Am Heart J 144: 1081–1086. 12486434 10.1067/mhj.2002.126737

[43] Nelson SD, Sparks EA, Graber HL, Boudoulas H, Mehdirad AA, Baker P, et al (1998) Clinical characteristics of sudden death victims in heritable (chromosome 1p1-1q1) conduction and myocardial disease. J Am Coll Cardiol 32: 1717–1723. 9822101 10.1016/s0735-1097(98)00424-0

[44] Kelly M, Semsarian C (2009) Multiple mutations in genetic cardiovascular disease: a marker of disease severity? Circ Cardiovasc Genet 2: 182–190. 10.1161/CIRCGENETICS.108.836478 20031583

[45] Dipple KM, McCabe ER (2000) Modifier genes convert "simple" Mendelian disorders to complex traits. Mol Genet Metab 71: 43–50. 11001794 10.1006/mgme.2000.3052

[46] Cooper DN, Krawczak M, Polychronakos C, Tyler-Smith C, Kehrer-Sawatzki H (2013) Where genotype is not predictive of phenotype: towards an understanding of the molecular basis of reduced penetrance in human inherited disease. Human Genetics 132: 1077–1130. 10.1007/s00439-013-1331-2 23820649 PMC3778950

[47] Nadeau JH (2003) Modifier genes and protective alleles in humans and mice. Current Opinion in Genetics and Development 13: 290–295. 12787792 10.1016/s0959-437x(03)00061-3

[48] Genin E, Feingold J, Clerget-Darpoux F (2008) Identifying modifier genes of monogenic disease: strategies and difficulties. Human Genetics 124: 357–368. 10.1007/s00439-008-0560-2 18784943 PMC2911473

[49] Brunham LR, Hayden MR (2013) Hunting human disease genes: lessons from the past, challenges for the future. Human Genetics 132: 603–617. 10.1007/s00439-013-1286-3 23504071 PMC3654184

[50] Mercuri E, Brown SC, Nihoyannopoulos P, Poulton J, Kinali M, Richard P, et al (2005) Extreme variability of skeletal and cardiac muscle involvement in patients with mutations in exon 11 of the lamin A/C gene. Muscle Nerve 31: 602–609. 15770669 10.1002/mus.20293

[51] Muntoni F, Bonne G, Goldfarb LG, Mercuri E, Piercy RJ, Burke M, et al (2006) Disease severity in dominant Emery Dreifuss is increased by mutations in both emerin and desmin proteins. Brain 129: 1260–1268. 16585054 10.1093/brain/awl062

[52] Murphy LL, Moon-Grady AJ, Cuneo BF, Wakai RT, Yu S, Kunic JD, et al (2012) Developmentally regulated SCN5A splice variant potentiates dysfunction of a novel mutation associated with severe fetal arrhythmia. Heart Rhythm 9: 590–597. 10.1016/j.hrthm.2011.11.006 22064211 PMC3292693

[53] Xu T, Yang Z, Vatta M, Rampazzo A, Beffagna G, Pilichou K, et al (2010) Compound and digenic heterozygosity contributes to arrhythmogenic right ventricular cardiomyopathy. J Am Coll Cardiol 55: 587–597. 10.1016/j.jacc.2009.11.020 20152563 PMC2852685

[54] Abecasis GR, Auton A, Brooks LD, DePristo MA, Durbin RM, Handsaker RE, et al (2012) An integrated map of genetic variation from 1,092 human genomes. Nature 491: 56–65. 10.1038/nature11632 23128226 PMC3498066

[55] Lupski JR, Belmont JW, Boerwinkle E, Gibbs RA (2011) Clan Genomics and the Complex Architecture of Human Disease. Cell 147: 32–43. 10.1016/j.cell.2011.09.008 21962505 PMC3656718

[56] Norton N, Li D, Rampersaud E, Morales A, Martin ER, Zuchner S, et al (2013) Exome sequencing and genome-wide linkage analysis in 17 families illustrate the complex contribution of TTN truncating variants to dilated cardiomyopathy. Circ Cardiovasc Genet 6: 144–153. 10.1161/CIRCGENETICS.111.000062 23418287 PMC3815606

[57] Lopes LR, Zekavati A, Syrris P, Hubank M, Giambartolomei C, Dalageorgou C, et al (2013) Genetic complexity in hypertrophic cardiomyopathy revealed by high-throughput sequencing. J Med Genet 50: 228–239. 10.1136/jmedgenet-2012-101270 23396983 PMC3607113

