# Supplementary material for: Exome Sequencing Identifies a Novel LMNA Splice-Site Mutation and Multigenic Heterozygosity of Potential Modifiers in a Family with Sick Sinus Syndrome, Dilated Cardiomyopathy, and Sudden Cardiac Death
Source: PLoS One. 2016 May 16;11(5):e0155421. doi: 10.1371/journal.pone.0155421 (PMC4868298; doi:10.1371/journal.pone.0155421)
Supplement: S1 File — (DOCX) [file pone.0155421.s001.docx]

**File S1: Supplementary materials**

**Table A: *LMNA* primers**

| **PCR/Sequencing Primers** | **Sequence (5’-3’)** |
| --- | --- |
| **Genomic DNA** |  |
| LMNA _exon 1_F | CCCAGATCCCGAGGTCCGAC |
| LMNA _exon 1_R | CCTCTCCACTCCCCGCCA |
| LMNA _exon 2_F | GGATGCCCTCTCCTGGTAAT |
| LMNA _exon 2_R | GCTCTGAAATCAGGTGACAGG |
| LMNA _exon 3_F | CCTTCCAGTTCTTGTGTTCTGTGAC |
| LMNA_exon 3_R | CCTAGCCCAGCCCAAGTCTGTC |
| LMNA_exon 4_F | GGCCTCCCAGGAACTAATTCTG |
| LMNA_exon 4_R | CTCCCTGCCACCATCTGC |
| LMNA _exon 5_F | GCTGTAGCAGTGATGCCCAAC |
| LMNA­_exon 5_R | CCAAAGCCCTGAGAAGTGAAG |
| LMNA _exon 6_F | GCCAAGACTATGTTTAGAGCTTG |
| LMNA _exon 6_R | GGTAGGGCTGGGGGACAG** |
| LMNA _exon 7_F | CCCCACTTGGTCTCCCTCTCC |
| LMNA _exon 7_R | CCCTGATGCAGCTGTATCCCC |
| LMNA _exon 8_F | GAGGCCTCAATTGCAGGCAGGC |
| LMNA_exon 8_R | GAAAAGGACACTTACCCCAGC |
| LMNA_exon 9_F | GGAGCGCTGGGGTAAGTGTC |
| LMNA_exon 9_R | CTCGTCCAGCAAGCAGCCAG |
| LMNA _exon 10_F | GTAAGCAGCAGGCCGGACAAAG |
| LMNA _exon 10_R | CACAGGAATATTCCATGGCATC |
| LMNA_exon 11_F | AGTGGTCAGTCCCAGACTCG |
| LMNA_exon 11_R | CCTGCAGGATTTGGAGACA |
| LMNA _exon 12_F | CTTGTCTGAGCCCCAGACTGGAG |
| LMNA _exon 12_R | AGGGAAAAGGAAGGGAGGAGAAAT |
|  |  |
| **cDNA** |  |
| **Exons 1-4** |  |
| LMNA cDNA Exon 1 F | GTGACTCAGTGTTCGCGGG |
| LMNA cDNA Exon 4 R | TCCACCAGTCGGGTCTCAT |
| **Exons 1-10** |  |
| LMNA cDNA Exon 1 Long F | CGTCGGTGACTCAGTGTTCG |
| LMNA cDNA Exon 10 Long R | CATCCTCGTCGTCCTCAACC |
| LMNA cDNA Exon 1-10 Internal F* | ATGAGACCCGACTGGTGGA |
| LMNA cDNA Exon 1-10 Internal R* | TCCTCATGCTGGGCCCGCAGT |
| **Exons 10-11** |  |
| LMNA cDNA Exon 10 F | GCGCTCAGTGACTGTGGTTG |
| LMNA cDNA Exon 11 R | GTGACCAGATTGTCCCCGAA |

*Primer used for sequencing only**Table B: Mean read coverage of each exon**

| **Genes** | **Number of exons** | **Total size (bases)** | **Mean coverage** | **Range: low** | **Range: high** |
| --- | --- | --- | --- | --- | --- |
| SeqCap EZ v2.0 (n=19,056) | 194,954 | 35,587,808 | 128.9 | 0.0 | 7107.7 |
| Candidate list (n=283) | 3,992 | 794,272 | 108.5 | 0.0 | 1989.8 |
| *AARS2* | 22 | 2,984 | 61.9 | 4.2 | 128.6 |
| *ABCC9* | 39 | 5,071 | 140.6 | 68.9 | 271.2 |
| *ACAD9* | 18 | 2,041 | 96.1 | 22.4 | 200.9 |
| *ACADS* | 10 | 1,310 | 47.8 | 0.7 | 134.3 |
| *ACADVL* | 20 | 2,284 | 86.5 | 7.9 | 168.7 |
| *ACTC1* | 6 | 1,134 | 126.2 | 28.2 | 184.6 |
| *ACTN2* | 21 | 2,806 | 129.2 | 36.4 | 284.2 |
| *AGL* | 34 | 4,764 | 175.9 | 46.9 | 373.9 |
| *AKAP9* | 51 | 12,191 | 106.3 | 26.8 | 259.5 |
| *ALG6* | 14 | 1,723 | 218.0 | 70.5 | 336.3 |
| *ALMS1* | 26 | 12,962 | 132.5 | 10.5 | 286.8 |
| *ANK2* | 48 | 12,469 | 138.1 | 37.1 | 508.9 |
| *ANKRD1* | 9 | 1,033 | 161.4 | 48.9 | 303.4 |
| *APOA1* | 3 | 861 | 46.7 | 31.6 | 56.5 |
| *BAG3* | 4 | 1,728 | 46.5 | 18.1 | 94.7 |
| *BIN1* | 20 | 2,123 | 99.8 | 10.6 | 279.0 |
| *BRAF* | 18 | 2,327 | 146.8 | 0.2 | 324.4 |
| *BRCC3* | 11 | 1,227 | 96.8 | 47.1 | 179.8 |
| *C2orf64* | 3 | 299 | 139.1 | 18.1 | 279.1 |
| *CACNA1A* | 49 | 7,343 | 98.1 | 0.2 | 290.3 |
| *CACNA1B* | 47 | 6,957 | 149.5 | 2.1 | 605.8 |
| *CACNA1C* | 52 | 7,558 | 142.9 | 16.5 | 702.8 |
| *CACNA1D* | 50 | 7,160 | 174.5 | 33.8 | 691.2 |
| *CACNA1E* | 47 | 7,140 | 181.5 | 27.4 | 531.0 |
| *CACNA1F* | 49 | 6,581 | 64.2 | 9.3 | 173.4 |
| *CACNA1G* | 38 | 7,599 | 117.3 | 8.3 | 431.3 |
| *CACNA1H* | 34 | 6,910 | 90.1 | 1.1 | 441.7 |
| *CACNA1I* | 37 | 6,134 | 110.8 | 0.0 | 431.0 |
| *CACNA1S* | 44 | 5,902 | 110.9 | 34.8 | 242.7 |
| *CACNA2D1* | 39 | 4,160 | 149.0 | 21.2 | 285.4 |
| *CACNA2D2* | 38 | 3,994 | 100.5 | 0.0 | 288.7 |
| *CACNA2D3* | 38 | 4,086 | 162.3 | 2.1 | 384.4 |
| *CACNA2D4* | 38 | 4,217 | 111.7 | 16.6 | 366.2 |
| *CACNB1* | 15 | 2,255 | 82.7 | 19.8 | 183.4 |
| *CACNB2* | 20 | 2,681 | 136.4 | 8.6 | 282.8 |
| *CACNB3* | 13 | 1,690 | 61.7 | 16.4 | 109.3 |
| *CACNB4* | 16 | 1,989 | 143.4 | 11.4 | 254.8 |
| *CACNG1* | 4 | 694 | 105.0 | 30.3 | 145.2 |
| *CACNG2* | 4 | 988 | 109.1 | 46.3 | 144.2 |
| *CACNG3* | 4 | 964 | 115.8 | 92.4 | 171.4 |
| *CACNG4* | 4 | 1,000 | 60.4 | 13.3 | 90.4 |
| *CACNG5* | 4 | 1,163 | 120.3 | 67.6 | 226.3 |
| *CACNG6* | 4 | 808 | 77.2 | 4.7 | 158.7 |
| *CACNG7* | 5 | 842 | 119.2 | 42.0 | 227.3 |
| *CACNG8* | 4 | 951 | 48.1 | 7.4 | 120.6 |
| *CALR3* | 9 | 1,179 | 174.8 | 36.0 | 341.0 |
| *CASQ2* | 11 | 1,366 | 150.2 | 70.3 | 329.1 |
| *CAV3* | 2 | 456 | 102.5 | 50.0 | 154.9 |
| *CDKN1C* | 2 | 951 | 7.2 | 4.9 | 9.4 |
| *CHRM2* | 1 | 1,401 | 160.8 | 160.8 | 160.8 |
| *CNBP* | 4 | 548 | 105.1 | 83.6 | 117.0 |
| *COQ9* | 9 | 1,110 | 91.3 | 12.5 | 207.9 |
| *COX10* | 7 | 1,418 | 143.1 | 33.7 | 264.9 |
| *COX15* | 10 | 1,361 | 128.2 | 41.2 | 416.2 |
| *CPT2* | 5 | 1,975 | 90.9 | 1.7 | 154.9 |
| *CRYAB* | 3 | 528 | 108.6 | 96.3 | 120.6 |
| *CSRP3* | 5 | 614 | 141.2 | 59.5 | 209.6 |
| *CTF1* | 3 | 219 | 45.7 | 0.0 | 89.8 |
| *CTSA* | 15 | 1,709 | 122.8 | 15.3 | 352.5 |
| *DES* | 9 | 1,588 | 69.1 | 11.6 | 119.7 |
| *DMD* | 87 | 12,486 | 109.3 | 2.2 | 357.3 |
| *DMPK* | 15 | 2,192 | 60.3 | 4.6 | 291.6 |
| *DNAJC19* | 6 | 600 | 170.2 | 18.4 | 267.0 |
| *DSC2* | 32 | 6,256 | 131.7 | 0.7 | 341.5 |
| *DSG2* | 15 | 3,437 | 98.4 | 0.7 | 193.1 |
| *DSP* | 24 | 8,685 | 115.4 | 12.2 | 290.7 |
| *DTNA* | 23 | 2,765 | 159.7 | 49.7 | 387.2 |
| *EMD* | 6 | 855 | 34.4 | 11.0 | 68.4 |
| *EYA4* | 20 | 2,317 | 169.6 | 93.2 | 319.0 |
| *FAH* | 14 | 1,486 | 147.1 | 37.0 | 220.8 |
| *FGA* | 6 | 2,691 | 177.2 | 93.7 | 376.6 |
| *FKRP* | 1 | 1,187 | 9.9 | 9.9 | 9.9 |
| *FKTN* | 9 | 1,426 | 112.5 | 57.1 | 165.0 |
| *FXN* | 6 | 654 | 167.6 | 0.5 | 304.5 |
| *GAA* | 19 | 2,919 | 69.6 | 12.8 | 375.5 |
| *GATA4* | 6 | 1,022 | 61.3 | 1.0 | 97.1 |
| *GBE1* | 16 | 2,151 | 145.3 | 40.7 | 296.3 |
| *GJA1* | 1 | 1,149 | 130.6 | 130.6 | 130.6 |
| *GJA5* | 1 | 1,077 | 116.9 | 116.9 | 116.9 |
| *GLA* | 7 | 1,298 | 75.5 | 46.5 | 119.5 |
| *GLB1* | 16 | 2,244 | 106.3 | 39.7 | 191.2 |
| *GNAI2* | 9 | 1,225 | 101.1 | 30.1 | 265.4 |
| *GNPTAB* | 21 | 3,924 | 177.6 | 37.7 | 394.4 |
| *GPD1L* | 8 | 1,113 | 99.5 | 17.1 | 260.1 |
| *GYG1* | 8 | 1,182 | 126.1 | 1.4 | 258.9 |
| *GYS1* | 16 | 2,299 | 80.6 | 18.6 | 192.3 |
| *HADHA* | 20 | 2,500 | 153.8 | 51.0 | 290.4 |
| *HADHB* | 15 | 1,756 | 170.1 | 65.0 | 378.8 |
| *HAMP* | 3 | 305 | 92.1 | 52.7 | 138.6 |
| *HCN4* | 8 | 3,439 | 73.3 | 14.8 | 125.6 |
| *HFE* | 6 | 1,130 | 173.1 | 27.4 | 529.4 |
| *HFE2* | 3 | 1,285 | 88.4 | 51.8 | 120.0 |
| *HRAS* | 5 | 670 | 60.0 | 24.9 | 115.6 |
| *ILK* | 12 | 1,409 | 113.5 | 45.1 | 158.6 |
| *JPH2* | 6 | 1,837 | 89.9 | 14.3 | 266.6 |
| *JUP* | 13 | 2,298 | 74.1 | 24.9 | 124.9 |
| *KCNA1* | 1 | 1,488 | 62.7 | 62.7 | 62.7 |
| *KCNA10* | 1 | 1,536 | 137.5 | 137.5 | 137.5 |
| *KCNA2* | 1 | 1,500 | 153.7 | 153.7 | 153.7 |
| *KCNA3* | 1 | 1,613 | 44.7 | 44.7 | 44.7 |
| *KCNA4* | 1 | 1,962 | 69.4 | 69.4 | 69.4 |
| *KCNA5* | 1 | 1,842 | 64.1 | 64.1 | 64.1 |
| *KCNA6* | 1 | 1,590 | 83.7 | 83.7 | 83.7 |
| *KCNA7* | 2 | 1,217 | 33.7 | 2.9 | 64.5 |
| *KCNAB1* | 16 | 2,083 | 161.0 | 44.4 | 294.6 |
| *KCNAB2* | 15 | 1,545 | 105.7 | 25.1 | 319.2 |
| *KCNAB3* | 14 | 1,575 | 128.9 | 2.0 | 208.1 |
| *KCNB1* | 2 | 2,577 | 87.9 | 71.5 | 104.2 |
| *KCNB2* | 2 | 2,736 | 68.9 | 63.1 | 74.7 |
| *KCNC1* | 4 | 1,826 | 81.3 | 18.1 | 146.3 |
| *KCNC2* | 5 | 2,017 | 135.9 | 14.8 | 197.6 |
| *KCNC3* | 4 | 1,732 | 34.7 | 4.8 | 71.8 |
| *KCNC4* | 5 | 2,091 | 98.6 | 28 | 156.4 |
| *KCND1* | 6 | 1,960 | 55.7 | 14.5 | 93.3 |
| *KCND2* | 11 | 2,408 | 137.2 | 81.6 | 246.8 |
| *KCND3* | 7 | 2,022 | 157.0 | 54.1 | 333.3 |
| *KCNE1* | 1 | 390 | 35 | 35 | 35.0 |
| *KCNE1L* | 1 | 367 | 3 | 3 | 3.0 |
| *KCNE2* | 1 | 372 | 126.8 | 126.8 | 126.8 |
| *KCNE3* | 1 | 312 | 47.1 | 47.1 | 47.1 |
| *KCNE4* | 1 | 513 | 35.1 | 35.1 | 35.1 |
| *KCNF1* | 1 | 1,485 | 52.9 | 52.9 | 52.9 |
| *KCNG1* | 2 | 1,595 | 32.4 | 30.9 | 33.9 |
| *KCNG2* | 2 | 1,228 | 13.8 | 4.4 | 23.1 |
| *KCNG3* | 2 | 1,304 | 46.7 | 8.2 | 85.2 |
| *KCNG4* | 2 | 1,574 | 42.3 | 28 | 56.6 |
| *KCNH1* | 11 | 2,991 | 145.7 | 51.4 | 295.1 |
| *KCNH2* | 16 | 3,535 | 44.9 | 0.8 | 137.6 |
| *KCNH3* | 15 | 3,299 | 51.7 | 9.5 | 167.8 |
| *KCNH4* | 16 | 3,165 | 65.8 | 20.5 | 215.4 |
| *KCNH5* | 12 | 3,094 | 132.6 | 61.8 | 223.6 |
| *KCNH6* | 14 | 3,025 | 64.9 | 11.1 | 109.6 |
| *KCNH7* | 16 | 3,764 | 176.1 | 78.1 | 304.9 |
| *KCNH8* | 16 | 3,432 | 136.8 | 63.5 | 243.8 |
| *KCNIP1* | 10 | 1,038 | 145.7 | 43.4 | 304.5 |
| *KCNIP2* | 11 | 1,170 | 78.0 | 18.3 | 187.0 |
| *KCNIP3* | 10 | 1,207 | 93.9 | 19.6 | 218.5 |
| *KCNIP4* | 11 | 1,143 | 163.1 | 57 | 383.5 |
| *KCNJ1* | 2 | 1,240 | 142.8 | 84.6 | 201.0 |
| *KCNJ10* | 1 | 1,140 | 144.2 | 144.2 | 144.2 |
| *KCNJ11* | 1 | 1,173 | 92.1 | 92.1 | 92.1 |
| *KCNJ12* | 1 | 1,302 | 86.8 | 86.8 | 86.8 |
| *KCNJ13* | 2 | 1,083 | 167.9 | 141.8 | 193.9 |
| *KCNJ14* | 2 | 1,311 | 59.9 | 11.1 | 108.6 |
| *KCNJ15* | 1 | 1,128 | 71.8 | 71.8 | 71.8 |
| *KCNJ16* | 1 | 1,257 | 141.8 | 141.8 | 141.8 |
| *KCNJ2* | 1 | 1,284 | 114.1 | 114.1 | 114.1 |
| *KCNJ3* | 3 | 1,506 | 113.7 | 101.3 | 134.8 |
| *KCNJ4* | 1 | 1,338 | 53 | 53 | 53.0 |
| *KCNJ5* | 2 | 1,260 | 70.7 | 36.8 | 104.6 |
| *KCNJ6* | 3 | 1,348 | 249.3 | 126.8 | 364.9 |
| *KCNJ8* | 2 | 1,275 | 111.6 | 105.4 | 117.7 |
| *KCNJ9* | 2 | 1,156 | 15.7 | 14.1 | 17.2 |
| *KCNK1* | 3 | 1,011 | 106.6 | 32.1 | 188.7 |
| *KCNK10* | 9 | 1,880 | 139.2 | 52.2 | 294.0 |
| *KCNK12* | 2 | 1,053 | 6.8 | 1.4 | 12.2 |
| *KCNK13* | 2 | 1,227 | 49.9 | 5.9 | 93.9 |
| *KCNK15* | 2 | 994 | 31.8 | 22.9 | 40.6 |
| *KCNK16* | 6 | 1,315 | 72.0 | 32.3 | 179.3 |
| *KCNK17* | 6 | 1,066 | 106.8 | 8.0 | 331.8 |
| *KCNK18* | 3 | 1,155 | 108.9 | 57.5 | 188.2 |
| *KCNK2* | 9 | 1,535 | 161.6 | 94.8 | 279.2 |
| *KCNK3* | 2 | 1,185 | 24.5 | 5.4 | 43.6 |
| *KCNK4* | 6 | 1,038 | 39.6 | 3.4 | 61.4 |
| *KCNK5* | 5 | 1,500 | 108.0 | 45.9 | 252.3 |
| *KCNK6* | 3 | 871 | 34.7 | 5.9 | 55.1 |
| *KCNK7* | 4 | 1,053 | 53.0 | 11.4 | 123.7 |
| *KCNK9* | 2 | 1,125 | 87.7 | 67 | 108.4 |
| *KCNMA1* | 31 | 4,216 | 137.4 | 24.6 | 365.1 |
| *KCNMB1* | 3 | 576 | 113.8 | 78.5 | 166.9 |
| *KCNMB2* | 4 | 752 | 90.5 | 68.2 | 100.9 |
| *KCNMB3* | 7 | 1,228 | 149.8 | 76.4 | 283.0 |
| *KCNMB4* | 3 | 633 | 123.9 | 33.1 | 171.3 |
| *KCNN1* | 9 | 1,719 | 82.6 | 7.4 | 160.2 |
| *KCNN2* | 8 | 1,774 | 130.1 | 19.6 | 285.7 |
| *KCNN3* | 9 | 2,330 | 162.1 | 66.5 | 391.0 |
| *KCNN4* | 8 | 1,286 | 96.4 | 13.3 | 222.3 |
| *KCNQ1* | 20 | 2,852 | 105.2 | 4.0 | 272.3 |
| *KCNQ1DN* | 2 | 267 | 91.5 | 52 | 131.0 |
| *KCNQ2* | 17 | 2,914 | 53.3 | 8.3 | 119.8 |
| *KCNQ3* | 22 | 3,973 | 127.6 | 37.1 | 331.6 |
| *KCNQ4* | 14 | 1,918 | 58.6 | 1.0 | 184.7 |
| *KCNQ5* | 15 | 2,922 | 192.1 | 7.4 | 520.2 |
| *KCNRG* | 2 | 819 | 104.5 | 75.6 | 133.4 |
| *KCNS1* | 3 | 1,543 | 58.6 | 12.1 | 95.4 |
| *KCNS2* | 1 | 1,434 | 108.5 | 108.5 | 108.5 |
| *KCNS3* | 1 | 1,476 | 103.3 | 103.3 | 103.3 |
| *KCNT1* | 32 | 4,067 | 64.0 | 1.3 | 233.9 |
| *KCNT2* | 28 | 3,668 | 135.7 | 35.4 | 372.9 |
| *KCNU1* | 29 | 4,113 | 180.8 | 57.9 | 360.5 |
| *KCNV1* | 3 | 1,503 | 76.1 | 21.0 | 105.9 |
| *KCNV2* | 2 | 1,638 | 83.3 | 34.9 | 131.7 |
| *KRAS* | 5 | 687 | 200.5 | 113.1 | 351.0 |
| *LAMA4* | 43 | 6,702 | 162.5 | 44.7 | 527.0 |
| *LAMP2* | 11 | 1,588 | 80.2 | 17.7 | 132.5 |
| *LDB3* | 16 | 2,695 | 87.0 | 18.2 | 225.9 |
| *LMNA* | 12 | 2,089 | 53.1 | 13.5 | 93.8 |
| *MAP2K1* | 11 | 1,399 | 164.5 | 39.1 | 264.1 |
| *MAP2K2* | 11 | 1,408 | 59.5 | 7.9 | 130.0 |
| *MLYCD* | 5 | 1,405 | 72.5 | 2.6 | 171.4 |
| *MRPS22* | 8 | 1,103 | 115.4 | 40 | 230.0 |
| *MYBPC3* | 34 | 4,518 | 90.6 | 15.1 | 271.4 |
| *MYH6* | 37 | 6,067 | 160.6 | 46.2 | 516.6 |
| *MYH7* | 38 | 6,064 | 155.6 | 39.3 | 478.2 |
| *MYL2* | 7 | 704 | 132.6 | 67.4 | 188.4 |
| *MYL3* | 6 | 753 | 187.1 | 66.5 | 396.1 |
| *MYLK2* | 12 | 1,894 | 69.7 | 26.7 | 135.3 |
| *MYO6* | 34 | 4,343 | 128.9 | 51.8 | 228.4 |
| *MYOT* | 9 | 1,547 | 97.5 | 48.3 | 182.8 |
| *MYOZ2* | 5 | 819 | 121.0 | 109.6 | 141.2 |
| *MYPN* | 19 | 4,133 | 111.2 | 49.9 | 179.2 |
| *NAA10* | 8 | 928 | 62.0 | 0.8 | 116.1 |
| *NDUFA2* | 3 | 308 | 93.5 | 27.7 | 142.1 |
| *NDUFS2* | 14 | 1,584 | 123.3 | 54.9 | 231.8 |
| *NDUFS8* | 6 | 734 | 56.9 | 30.3 | 82.8 |
| *NDUFV2* | 8 | 896 | 96.4 | 16.4 | 152.8 |
| *NEBL* | 32 | 3,551 | 206.3 | 37 | 438.8 |
| *NEXN* | 12 | 2,182 | 80.1 | 27.3 | 132.4 |
| *NKX2-5* | 3 | 1,181 | 24.9 | 21.4 | 27.3 |
| *NPPA* | 3 | 550 | 104.5 | 77.5 | 120.8 |
| *NSD1* | 22 | 8,115 | 121.8 | 41 | 243.4 |
| *PKP2* | 14 | 2,689 | 158.1 | 7 | 389.4 |
| *PKP4* | 21 | 3,686 | 116.1 | 32.6 | 224.5 |
| *PLN* | 1 | 159 | 195.8 | 195.8 | 195.8 |
| *PMM2* | 8 | 830 | 124.1 | 20.7 | 225.9 |
| *PNPLA2* | 9 | 1,466 | 44.3 | 2.7 | 109.6 |
| *POLG* | 22 | 3,816 | 88.2 | 13.1 | 251.4 |
| *PRKAG2* | 54 | 13,540 | 171.6 | 1.9 | 1989.8 |
| *PSEN1* | 10 | 1,464 | 185.6 | 94.9 | 469.5 |
| *PSEN2* | 10 | 1,395 | 114.4 | 27.9 | 313.1 |
| *PTPN11* | 15 | 1,954 | 124.0 | 11.5 | 264.5 |
| *PTRF* | 2 | 1,110 | 65.6 | 34.7 | 96.4 |
| *RAF1* | 16 | 2,088 | 133.8 | 74.1 | 272.3 |
| *RANGRF* | 5 | 730 | 72.6 | 23 | 116.9 |
| *RBM20* | 14 | 3,688 | 109.0 | 2.1 | 265.4 |
| *RPS6KA3* | 22 | 2,512 | 95.6 | 2.4 | 310.6 |
| *RYR2* | 105 | 15,974 | 164.5 | 15.1 | 553.5 |
| *SCN10A* | 27 | 5,978 | 130.7 | 68.3 | 237.7 |
| *SCN11A* | 26 | 5,462 | 147.5 | 52.2 | 327.1 |
| *SCN1A* | 26 | 6,130 | 175.0 | 87.5 | 376.9 |
| *SCN1B* | 5 | 1,009 | 64.8 | 0.0 | 98.6 |
| *SCN2A* | 27 | 6,218 | 188.3 | 35.9 | 314.9 |
| *SCN2B* | 4 | 678 | 168.5 | 60.5 | 302.0 |
| *SCN3A* | 27 | 6,203 | 180.8 | 66.8 | 174.9 |
| *SCN3B* | 5 | 729 | 103.1 | 67.8 | 167.9 |
| *SCN4A* | 24 | 5,611 | 116.6 | 20.8 | 285.2 |
| *SCN4B* | 5 | 732 | 141.8 | 29.0 | 307.0 |
| *SCN5A* | 28 | 6,251 | 106.6 | 11.5 | 241.0 |
| *SCN7A* | 24 | 5,147 | 131.0 | 33.9 | 367.5 |
| *SCN8A* | 26 | 6,043 | 172.8 | 15.3 | 460.8 |
| *SCN9A* | 27 | 6,043 | 159.6 | 76.8 | 378.7 |
| *SCNM1* | 7 | 797 | 127.2 | 69.6 | 205.1 |
| *SCNN1A* | 13 | 2,418 | 93.2 | 18.9 | 200.3 |
| *SCNN1B* | 12 | 2,051 | 89.4 | 49.9 | 163.8 |
| *SCNN1D* | 15 | 2,308 | 28.1 | 5.8 | 60.9 |
| *SCNN1G* | 12 | 2,075 | 89.4 | 45.1 | 123.6 |
| *SCO2* | 1 | 801 | 39.5 | 39.5 | 39.5 |
| *SDHA* | 15 | 2,060 | 128.2 | 1.0 | 229.3 |
| *SGCD* | 8 | 1,083 | 116.2 | 55.0 | 151.3 |
| *SLC22A5* | 10 | 1,686 | 175.2 | 21.4 | 328.8 |
| *SLC25A3* | 8 | 1,211 | 225.4 | 19.2 | 524.6 |
| *SLC25A4* | 4 | 897 | 97.8 | 25.5 | 142.3 |
| *SMC1A* | 25 | 3,766 | 123.6 | 31.6 | 367.2 |
| *SNTA1* | 8 | 1,216 | 53.6 | 0.0 | 112.7 |
| *SOS1* | 23 | 4,089 | 143.0 | 16.3 | 366.2 |
| *TAZ* | 11 | 1,141 | 35.4 | 5.9 | 64.3 |
| *TBX20* | 13 | 2,175 | 142.0 | 49.9 | 290.8 |
| *TBX5* | 8 | 1,639 | 150.2 | 47.4 | 303.6 |
| *TCAP* | 2 | 504 | 61.6 | 24.8 | 98.4 |
| *TGFB3* | 7 | 1,239 | 142.1 | 75.5 | 358.0 |
| *TMEM43* | 12 | 1,431 | 109.2 | 6.7 | 202.3 |
| *TMEM70* | 3 | 791 | 124.6 | 16.8 | 238.8 |
| *TMPO* | 10 | 2,903 | 112.0 | 19.0 | 202.1 |
| *TNNC1* | 6 | 699 | 133.0 | 37.4 | 287.6 |
| *TNNI3* | 8 | 912 | 104.8 | 5.2 | 326.2 |
| *TNNT2* | 18 | 1,846 | 145.8 | 59.1 | 610.2 |
| *TPM1* | 15 | 1,652 | 120.1 | 4.2 | 456.3 |
| *TRPM4* | 25 | 3,935 | 85.9 | 18.4 | 453.4 |
| *TSFM* | 6 | 1,033 | 121.2 | 53.0 | 265.6 |
| *TTN* | 315 | 111,761 | 181.7 | 53.0 | 709.3 |
| *TTR* | 4 | 475 | 133.8 | 64.2 | 211.1 |
| *VCL* | 22 | 3,444 | 92.3 | 14.9 | 227.2 |
| *VCP* | 17 | 2,404 | 148.3 | 0.0 | 360.5 |

**Appendix A: 283 candidate gene list**

**I. Known arrhythmia and cardiomyopathy associated genes in humans (n=158)**

| **Gene** | **Location^a^** | **Arrhythmia** | **Cardiomyopathy** | **Type^b^** | **References** |
| --- | --- | --- | --- | --- | --- |
| ***AARS2*** | 6p21.1 |  | x | mitochondrial-nuclear | Gotz 2011 |
| ***ABCC9*** | 12p12.1 | x | x | ion channel | Bienengraeber 2004 |
| ***ACAD9*** | 3q21.3 |  | x | metabolic disease | He 2007 |
| ***ACADS*** | 12q24.31 |  | x | metabolic disease | Tein 2008 |
| ***ACADVL*** | 17p13-p11 | x | x | metabolic disease | Mathur 1999 |
| ***ACTC1*** | 15q11-q14 |  | x | sarcomeric | Olson 1998 |
| ***ACTN2*** | 1q42-q43 |  | x | sarcomeric | Mohapatra 2003 |
| ***AGL*** | 1p21 |  | x | metabolic disease | Shen 1996 |
| ***AKAP9*** | 7q21.2 | x |  | signaling (protein kinase A) | Chen 2007 |
| ***ALG6*** | 1p31.3 |  | x | metabolic disease: CDG | Al-Owain 2010 |
| ***ALMS1*** | 2p13 |  | x | mitochondrial-nuclear | Collin 2002 |
| ***ANK2*** | 4q25 | x |  | cytoskeletal: membrane attachment | Mohler 2003 |
| ***ANKRD1; CARP*** | 10q23.31 |  | x | other: transcription factor | Arimura 2009 |
| ***APOA1*** | 11p11-q13 |  | x | metabolic disease | Hamidi Asl 1999 |
| ***BAG3*** | 10q26.11 |  | x | other: apoptosis | Selcen 2008 |
| ***BIN1*** | 2q14.3 | x |  | other: tumor supressor, adaptor | Bohm 2010 |
| ***BRAF*** | 7q34 | x | x | signaling (MAPK, ErbB) | Niihori 2006 |
| ***BRCC3*** | Xq28 |  | x | other: cell cycle and DNA damage | Miskinyte 2011 |
| ***C2orf64*** | 2q11.2 |  | x | mitochondrial-nuclear | Huigsloot 2011 |
| ***CACNA1C*** | 12p13.33 | x |  | ion channel: Ca++ | Splawski 2004 |
| ***CACNA1D*** | 3p21.1 | x |  | ion channel: Ca++ | Baig 2011 |
| ***CACNB2*** | 10p12.33 | x |  | ion channel: Ca++ | Antzelevitch 2007 |
| ***CALR3*** | 19p13.11 |  | x | other: Ca++ homeostasis | Chiu 2007 |
| ***CASQ2*** | 1p13.1 | x |  | other: Ca++ homeostasis | Lahat 2001 |
| ***CAV3*** | 3p25 | x | x | cytoskeletal | Hayashi 2004b; Vatta 2006 |
| ***CDKN1C*** | 11p15.5 |  | x | signaling (cell cycle) | Alizad 2000 |
| ***CHRM2; M2MR*** | 7q33 | x | x | signaling (Calcium) | Zhang 2008 |
| ***CNBP; DM2*** | 3q21.3 | x | x | other: DNA-binding protein | Day 2003; Schneider-Gold 2004 |
| ***COQ9*** | 16q13 |  | x | mitochondrial-nuclear | Duncan 2009 |
| ***COX10*** | 17p12 |  | x | mitochondrial-nuclear | Antonicka 2003a |
| ***COX15*** | 10q24 |  | x | mitochondrial-nuclear | Antonicka 2003b |
| ***CPT2*** | 1p32 |  | x | mitochondrial-nuclear | Thuillier 2003 |
| ***CRYAB*** | 11q23.1 | x | x | cytoskeletal | Vicart 1998; Arbustini 2006 |
| ***CSRP3; MLP*** | 11p15.1 |  | x | sarcomeric / Z-disk | Knoll 2002 |
| ***CTF1*** | 16p11.2 |  | x | signaling (Jak-sTAT) | Erdmann 2000 |
| ***CTSA; PPGB*** | 20q13.1 |  | x | metabolic disease | Sewell 1987; Guertl 2000 |
| ***DES*** | 2q35 | x | x | cytoskeletal | Li 1999 |
| ***DMD*** | Xp21.2 |  | x | cytoskeletal | Koenig 1987 |
| ***DMPK*** | 19q13.3 | x | x | other: Ca++ homeostasis | Phillips 1997; Sovari 2007 |
| ***DNAJC19*** | 3q26.33 | x | x | mitochondrial-nuclear | Davey 2006; Sparkes 2007 |
| ***DSC2*** | 18q12.1 | x | x | desmosomal | Syrris 2006; Heuser 2006 |
| ***DSG2*** | 18q12.1 | x | x | desmosomal | Awad 2006; Pilichou 2006 |
| ***DSP*** | 6p24 | x | x | desmosomal | Rampazzo 2002 |
| ***DTNA*** | 18q12.1 |  | x | cytoskeletal | Ichida 2001 |
| ***EMD*** | Xq28 | x | x | nuclear envelope | Sakata 2005 |
| ***EYA4*** | 6q23-q24 |  | x | other: transcription | Schonberger 2005 |
| ***FAH*** | 15q23-q25 |  | x | metabolic disease | Edwards 1987 |
| ***FGA*** | 4q32.1 |  | x | other: clotting | Mourad 2008 |
| ***FKRP*** | 19q13.32 |  | x | cytoskeletal | Muller 2005 |
| ***FKTN; FCMD*** | 9q31.2 |  | x | other: glycosylation | Murakami 2006 |
| ***FXN*** | 9q13-q21.1 |  | x | mitochondrial-nuclear | Durr 1996 |
| ***GAA*** | 17q25.2-q25.3 | x | x | metabolic disease | Pompe 1932; Suzuki 1988 |
| ***GATA4*** | 8p23.1 | x |  | other: transcription factor | Posch 2010 |
| ***GBE1*** | 3p12.2 |  | x | metabolic disease: glycogen | Bao 1996 |
| ***GJA1; Cx43*** | 6q22.31 | x |  | other: gap junction | Thibodeau 2010 |
| ***GJA5; Cx40*** | 1q21.2 | x |  | other: gap junction | Gollob 2006 |
| ***GLA*** | Xq22 |  | x | metabolic disease | Elleder 1990; Monserrat 2007 |
| ***GLB1*** | 3p22.3 |  | x | metabolic disease: storage disease | Brunetti-Pierri 2008 |
| ***GNAI2*** | 3p21.31 | x |  | signaling (G-protein) | Lerman 1998 |
| ***GNPTAB*** | 12q23.3 |  | x | metabolic disease | Guertl 2000 |
| ***GPD1L*** | 3p22.3 | x |  | signaling (sodium) | London 2007 |
| ***GYG1*** | 3q24 | x |  | metabolic disease: glycogen | Moslemi 2010 |
| ***GYS1*** | 19q13.33 |  | x | metabolic disease: glycogen | Kollberg 2007 |
| ***HADHA*** | 2p23 |  | x | mitochondrial-nuclear | Den Boer 2002 |
| ***HADHB*** | 2p23 |  | x | mitochondrial-nuclear | Spiekerkoetter 2003 |
| ***HAMP; HFE2B*** | 19q13 | x | x | metabolic disease | Roetto 2003; De Gobbi 2002 |
| ***HCN4*** | 15q24.1 | x |  | ion channel | Schulze-Bahr 2003; Milanesi 2006 |
| ***HFE*** | 6p21.3 | x | x | metabolic disease | Cutler 1980; Hannuksela 2005 |
| ***HFE2; HJV*** | 1q21.1 | x | x | metabolic disease | Papanikolaou 2004 (RCM) |
| ***HRAS*** | 11p15.5 | x | x | signaling (MAPK, ErbB) | Aoki 2005 |
| ***ILK*** | 11p15.4 |  | x | other: extracellular matrix | Knoll 2007 |
| ***JPH2*** | 20q13.12 |  | x | other: Ca++ homeostasis | Landstrom 2007 |
| ***JUP*** | 17q21 | x | x | desmosomal | McKoy 2000; Asimaki 2007 |
| ***KCNA5*** | 12p13.32 | x |  | ion channel: K+ | Olson 2006 |
| ***KCNE1*** | 21q22.12 | x |  | ion channel: K+ | Ohno 2007 |
| ***KCNE2*** | 21q22.11 | x |  | ion channel: K+ | Yang 2004 |
| ***KCNE3*** | 11q13.4 | x |  | ion channel: K+ | Delpon 2008 |
| ***KCNH2*** | 7q36.1 | x |  | ion channel: K+ | Brugada 2004 |
| ***KCNJ2*** | 17q24.3 | x |  | ion channel: K+ | Eckhardt 2007 |
| ***KCNJ5*** | 11q24.3 | x |  | ion channel: K+ | Yang 2010 |
| ***KCNJ8*** | 12p12.1 | x |  | ion channel: K+ | Haissaguerre 2009; Medeiros-Domingo 2010 |
| ***KCNQ1*** | 11p15.5 | x |  | ion channel: K+ | Wang 1996; Chen 2003 |
| ***KRAS*** | 12p12.1 | x | x | signaling (MAPK, ErbB) | Niihori 2006; Schubbert 2006 |
| ***LAMA4*** | 6q21 |  | x | other: extracellular matrix | Knoll 2007 |
| ***LAMP2*** | Xq24 |  | x | metabolic disease | Nishino 2000; Arad 2005 |
| ***LDB3; ZASP*** | 10q22.3-q23.2 |  | x | sarcomeric / Z-disk | Vatta 2003 |
| ***LMNA*** | 1q21.2-q21.3 | x | x | nuclear envelope | Fatkin 1999 |
| ***MAP2K1; MEK1*** | 15q22.31 | x | x | signaling (MAPK, ErbB) | Narumi 2007; Gripp 2007 |
| ***MAP2K2; MEK2*** | 19p13.3 | x | x | signaling (MAPK, ErbB) | Narumi 2007 |
| ***MLYCD*** | 16q24 |  | x | metabolic disease | FitzPatrick 1999 |
| ***MRPS22*** | 3q23 |  | x | mitochondrial-nuclear | Saada 2007 |
| ***MYBPC3*** | 11p11.2 |  | x | sarcomeric | Watkins 1995 |
| ***MYH6*** | 14q12 | x | x | sarcomeric and SNP-association | Tanigawa 1990; Holm 2011 |
| ***MYH7*** | 14q12 |  | x | sarcomeric | Geisterfer-Lowrance 1990 |
| ***MYL2*** | 12q23-q24.3 |  | x | sarcomeric | Poetter 1996 |
| ***MYL3*** | 3p21.3-p21.2 |  | x | sarcomeric | Poetter 1996 |
| ***MYLK2*** | 20q13.31 |  | x | cytoskeletal | Davis 2001 |
| ***MYO6*** | 6q13 |  | x | other: actin-based motor | Mohiddin 2004 |
| ***MYOT*** | 5q31 | x | x | sarcomeric / Z-disk | Selcen 2004 |
| ***MYOZ2*** | 4q26 |  | x | sarcomeric / Z-disk | Osio 2007 |
| ***MYPN*** | 10q21.1 |  | x | sarcomeric / Z-disk | Duboscq-Bidot 2008 |
| ***NAA10*** | Xq28 | x |  | other: protein modification | Rope 2011 |
| ***NDUFA2*** | 5q31.3 |  | x | mitochondrial-nuclear | Hoefs 2008 |
| ***NDUFS2*** | 1q23 |  | x | mitochondrial-nuclear | Loeffen 2001 |
| ***NDUFS8*** | 11q13 |  | x | mitochondrial-nuclear | Loeffen 1999 |
| ***NDUFV2*** | 18p11.31-p11.2 |  | x | mitochondrial-nuclear | Benit 2003 |
| ***NEBL*** | 10p12.31 |  | x | cytoskeletal and SNP-association | Purevjav 2010 |
| ***NEXN*** | 1p31.1 |  | x | cytoskeleton | Hassel 2009 |
| ***NKX2-5*** | 5q35.1 | x | x | other: transcription factor | Schott 1998; Ouyang 2011 |
| ***NPPA*** | 1p36.22 | x |  | signaling- volume homeostatsis | Hodgson-Zingman 2008 |
| ***NSD1*** | 5q35.2 |  | x | other: transcription, histone methyl | Martinez 2011 |
| ***PKP2*** | 12p11 | x | x | desmosomal | Gerull 2004 |
| ***PKP4*** | 2q24.1 | x | x | desmosomal | Xu 2010 |
| ***PLN*** | 6q22.1 |  | x | other: Ca++ homeostasis | Schmitt 2003 |
| ***PMM2*** | 16p13.3-p13.2 |  | x | metabolic disease | Clayton 1992 |
| ***PNPLA2*** | 11p15.5 |  | x | metabolic disease: lipid | Chen 2010 |
| ***POLG*** | 15q25 |  | x | mitochondrial-nuclear | Van Goethem 2004 |
| ***PRKAG2*** | 7q36 | x | x | metabolic disease | Blair 2001; Gollob 2001 |
| ***PSEN1*** | 14q24.2 |  | x | signaling | Li 2006 |
| ***PSEN2*** | 1q42.13 |  | x | signaling | Li 2006 |
| ***PTPN11*** | 12q24.13 | x | x | signaling (Jak-sTAT) | Tartaglia 2002; Limongelli 2008 |
| ***PTRF*** | 17q21.2 | x |  | other: transcription | Rajab 2010 |
| ***RAF1*** | 3p25.2 | x | x | signaling (MAPK, ErbB) | Pandit 2007; Limongelli 2008 |
| ***RANGRF; MOG1*** | 17p13.1 | x |  | other: trafficking (SCN5A) | Kattygnarath 2011 |
| ***RBM20*** | 10q25.2 |  | x | other: Unknown | Brauch 2009 |
| ***RPS6KA3; RSK2*** | Xp22.12 |  | x | signaling | Massin 1999 |
| ***RYR2*** | 1q42-q43 | x | x | ion channel: Ca++ | Tiso 2001 |
| ***SCN1B*** | 19q13.12 | x |  | ion channel: Na+ | Watanabe 2008 |
| ***SCN3B*** | 11q24.1 | x |  | ion channel: Na+ | Hu 2009 |
| ***SCN4B*** | 11q23.3 | x |  | ion channel: Na+ | Medeiros-Domingo 2007 |
| ***SCN5A*** | 3p25-p22 | x | x | ion channel: Na+ | Wang 1995; McNair 2004 |
| ***SCO2*** | 22q13.33 |  | x | mitochondrial-nuclear | Papadopoulou 1999 |
| ***SDHA*** | 5p15.33 |  | x | mitochondrial-nuclear | Levitas 2010 |
| ***SGCD*** | 5q33-q34 |  | x | cytoskeletal | Tsubata 2000 |
| ***SLC22A5; OCTN2*** | 5q31 |  | x | mitochondrial-nuclear/metabolic dis | Nezu 1999 |
| ***SLC25A3*** | 12q23.1 |  | x | mitochondrial-nuclear | Mayr 2007 |
| ***SLC25A4; ANT1*** | 4q35 |  | x | mitochondrial-nuclear | Palmieri 2005 |
| ***SMC1A*** | Xp11.22 |  | x | other: cell division | Limongelli 2010 |
| ***SNTA1*** | 20q11.21 | x |  | cytoskeletal: adapter protein | Ueda 2008 |
| ***SOS1*** | 2p22.1 |  | x | signaling (MAPK, ErbB) | Roberts 2007; Tartaglia 2007 |
| ***TAZ; G4.5*** | Xq28 |  | x | mitochondrial-nuclear | Bione 1996 |
| ***TBX20*** | 7p14.2 |  | x | other: DNA-binding protein | Kirk 2007 |
| ***TBX5*** | 12q24.21 | x |  | other: DNA-binding protein | Basson 1997 |
| ***TCAP*** | 17q12 |  | x | sarcomeric / Z-disk | Hayashi 2004a |
| ***TGFB3*** | 14q24.3 | x | x | signaling | Beffagna 2005 |
| ***TMEM43*** | 3p25.1 | x | x | other: membrane protein | Merner 2008 |
| ***TMEM70*** | 8q21.11 |  | x | mitochondrial-nuclear | Cízková 2008 |
| ***TMPO; LAP2*** | 12q23.1 |  | x | nuclear envelope | Taylor 2005 |
| ***TNNC1*** | 3p14.3-21.3 |  | x | sarcomeric | Hoffmann 2001 |
| ***TNNI3*** | 19q13.4 |  | x | sarcomeric | Kimura 1997 |
| ***TNNT2*** | 1q32 |  | x | sarcomeric | Thierfelder 1994 |
| ***TPM1*** | 15q22.1 |  | 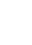x | sarcomeric | Thierfelder 1994 |
| ***TRPM4*** | 19q13.33 | x |  | ion channel | Kruse 2009 |
| ***TSFM*** | 12q14.1 |  | x | mitochondrial-nuclear | Smeitink 2006 |
| ***TTN*** | 2q31 |  | x | sarcomeric / Z-disk | Satoh 1999; Gerull 2002 |
| ***TTR*** | 18q12.1 | x | x | metabolic disease | Saraiva 1995; Mörner 2005 |
| ***VCL*** | 10q22.1-q23 |  | x | cytoskeletal | Olson 2002 |
| ***VCP*** | 9p13.3 |  | x | other: cell cycle, golgi assembly | Hübbers 2007 |

^a^Nuclear-encoded genes only

^b^Both syndromic and non-syndromic conditions are included.

**II. Other potassium, sodium or calcium ion channel genes (n=125 genes)**

| *CACNA1A* | *KCNA2* | *KCNH3* | *KCNK13* | *KCNQ5* |
| --- | --- | --- | --- | --- |
| *CACNA1B* | *KCNA3* | *KCNH4* | *KCNK15* | *KCNRG* |
| *CACNA1E* | *KCNA4* | *KCNH5* | *KCNK16* | *KCNS1* |
| *CACNA1F* | *KCNA6* | *KCNH6* | *KCNK17* | *KCNS2* |
| *CACNA1G* | *KCNA7* | *KCNH7* | *KCNK18* | *KCNS3* |
| *CACNA1H* | *KCNAB1* | *KCNH8* | *KCNK2* | *KCNT1* |
| *CACNA1I* | *KCNAB2* | *KCNIP1* | *KCNK3* | *KCNT2* |
| *CACNA1S* | *KCNAB3* | *KCNIP2* | *KCNK4* | *KCNU1* |
| *CACNA2D1* | *KCNB1* | *KCNIP3* | *KCNK5* | *KCNV1* |
| *CACNA2D2* | *KCNB2* | *KCNIP4* | *KCNK6* | *KCNV2* |
| *CACNA2D3* | *KCNC1* | *KCNJ1* | *KCNK7* | *SCN10A* |
| *CACNA2D4* | *KCNC2* | *KCNJ10* | *KCNK9* | *SCN11A* |
| *CACNB1* | *KCNC3* | *KCNJ11* | *KCNMA1* | *SCN1A* |
| *CACNB3* | *KCNC4* | *KCNJ12* | *KCNMB1* | *SCN2A* |
| *CACNB4* | *KCND1* | *KCNJ13* | *KCNMB2* | *SCN2B* |
| *CACNG1* | *KCND2* | *KCNJ14* | *KCNMB3* | *SCN3A* |
| *CACNG2* | *KCND3* | *KCNJ15* | *KCNMB4* | *SCN4A* |
| *CACNG3* | *KCNE1L* | *KCNJ16* | *KCNN1* | *SCN7A* |
| *CACNG4* | *KCNE4* | *KCNJ3* | *KCNN2* | *SCN8A* |
| *CACNG5* | *KCNF1* | *KCNJ4* | *KCNN3* | *SCN9A* |
| *CACNG6* | *KCNG1* | *KCNJ6* | *KCNN4* | *SCNM1* |
| *CACNG7* | *KCNG2* | *KCNJ9* | *KCNQ1DN* | *SCNN1A* |
| *CACNG8* | *KCNG3* | *KCNK1* | *KCNQ2* | *SCNN1B* |
| *KCNA1* | *KCNG4* | *KCNK10* | *KCNQ3* | *SCNN1D* |
| *KCNA10* | *KCNH1* | *KCNK12* | *KCNQ4* | *SCNN1G* |

**References**

Alizad A, Seward JB. Echocardiographic features of genetic diseases: part 1.Cardiomyopathy. *J Am Soc Echocardiogr*. 2000;**13**:73-86.

Al-Owain M, Mohamed S, Kaya N, Zagal A, Matthijs G, Jaeken J. A novel mutation and first report of dilated cardiomyopathy in ALG6-CDG (CDG-Ic): a case report. *Orphanet J Rare Dis*. 2010;**5**:7.

Antonicka H, Leary SC, Guercin GH, Agar JN, Horvath R, Kennaway NG, Harding CO, Jaksch M, Shoubridge EA. Mutations in COX10 result in a defect in mitochondrial heme A biosynthesis and account for multiple, early-onset clinical phenotypes associated with isolated COX deficiency. *Hum Mol Genet*. 2003a;**12**:2693-2702.

Antonicka H, Mattman A, Carlson CG, Glerum DM, Hoffbuhr KC, Leary SC, Kennaway NG, Shoubridge EA. Mutations in COX15 produce a defect in the mitochondrial heme biosynthetic pathway, causing early-onset fatal hypertrophic cardiomyopathy. *Am J Hum Genet*. 2003b;**72**:101-114.

Antzelevitch C, Pollevick GD, Cordeiro JM, Casis O, Sanguinetti MC, Aizawa Y, Guerchicoff A, Pfeiffer R, Oliva A, Wollnik B, Gelber P, Bonaros EP Jr, Burashnikov E, Wu Y, Sargent JD, Schickel S, Oberheiden R, Bhatia A, Hsu LF, Haïssaguerre M, Schimpf R, Borggrefe M, Wolpert C. Loss-of-function mutations in the cardiac calcium channel underlie a new clinical entity characterized by ST-segment elevation, short QT intervals, and sudden cardiac death. *Circulation*. 2007;**115**:442-449.

Aoki Y, Niihori T, Kawame H, Kurosawa K, Ohashi H, Tanaka Y, Filocamo M, Kato K, Suzuki Y, Kure S, Matsubara Y. Germline mutations in HRAS proto-oncogene cause Costello syndrome. *Nat Genet*. 2005;**37**:1038-1040.

Arad M, Maron BJ, Gorham JM, Johnson WH Jr, Saul JP, Perez-Atayde AR, Spirito P, Wright GB, Kanter RJ, Seidman CE, Seidman JG. Glycogen storage diseases presenting as hypertrophic cardiomyopathy. *N Engl J Med*. 2005;**352**:362-372.

Arbustini E, Pasotti M, Pilotto A, Pellegrini C, Grasso M, Previtali S, Repetto A, Bellini O, Azan G, Scaffino M, Campana C, Piccolo G, Viganò M, Tavazzi L. Desmin accumulation restrictive cardiomyopathy and atrioventricular block associated with desmin gene defects. *Eur J Heart Fail*. 2006;**8**:477-483.

Arimura T, Bos JM, Sato A, Kubo T, Okamoto H, Nishi H, Harada H, Koga Y, Moulik M, Doi YL, Towbin JA, Ackerman MJ, Kimura A. Cardiac ankyrin repeat protein gene (ANKRD1) mutations in hypertrophic cardiomyopathy. *J Am Coll Cardiol*. 2009;**54**:334-342.

Asimaki A, Syrris P, Wichter T, Matthias P, Saffitz JE, McKenna WJ. A novel dominant mutation in plakoglobin causes arrhythmogenic right ventricular cardiomyopathy. *Am J Hum Genet*. 2007;**81**:964-973.

Awad MM, Dalal D, Cho E, Amat-Alarcon N, James C, Tichnell C, Tucker A, Russell SD, Bluemke DA, Dietz HC, Calkins H, Judge DP. DSG2 mutations contribute to arrhythmogenic right ventricular dysplasia/cardiomyopathy. *Am J Hum Genet*. 2006;**79**:136–142.

Baig SM, Koschak A, Lieb A, Gebhart M, Dafinger C, Nürnberg G, Ali A, Ahmad I, Sinnegger-Brauns MJ, Brandt N, Engel J, Mangoni ME, Farooq M, Khan HU, Nürnberg P, Striessnig J, Bolz HJ. Loss of Ca(v)1.3 (CACNA1D) function in a human channelopathy with bradycardia and congenital deafness. *Nat Neurosci*. 2011;**14**:77-84.

Bao Y, Kishnani P, Wu JY, Chen YT. Hepatic and neuromuscular forms of glycogen storage disease type IV caused by mutations in the same glycogen-branching enzyme gene. *J Clin Invest*. 1996;**97**:941-948.

Basson CT, Bachinsky DR, Lin RC, Levi T, Elkins JA, Soults J, Grayzel D, Kroumpouzou E, Traill TA, Leblanc-Straceski J, Renault B, Kucherlapati R, Seidman JG, Seidman CE. Mutations in human TBX5 [corrected] cause limb and cardiac malformation in Holt-Oram syndrome. *Nat Genet*. 1997;**15**:30-35.

Beffagna G, Occhi G, Nava A, Vitiello L, Ditadi A, Basso C, Bauce B, Carraro G, Thiene G, Towbin JA, Danieli GA, Rampazzo A. Regulatory mutations in transforming growth factor-beta3 gene cause arrhythmogenic right ventricular cardiomyopathy type 1. *Cardiovasc Res*. 2005;**65**:366-373.

Bénit P, Beugnot R, Chretien D, Giurgea I, De Lonlay-Debeney P, Issartel JP, Corral-Debrinski M, Kerscher S, Rustin P, Rötig A, Munnich A. Mutant NDUFV2 subunit of mitochondrial complex I causes early onset hypertrophic cardiomyopathy and encephalopathy. *Hum Mutat*. 2003;**21**:582-586.

Bienengraeber M, Olson TM, Selivanov VA, Kathmann EC, O'Cochlain F, Gao F, Karger AB, Ballew JD, Hodgson DM, Zingman LV, Pang YP, Alekseev AE, Terzic A. ABCC9 mutations identified in human dilated cardiomyopathy disrupt catalytic KATP channel gating. *Nat Genet*. 2004;**36**:382-387.

Bione S, D'Adamo P, Maestrini E, Gedeon AK, Bolhuis PA, Toniolo D. A novel X-linked gene, G4.5. is responsible for Barth syndrome. *Nat Genet*. 1996;**12**:385-389.

Blair E, Redwood C, Ashrafian H, Oliveira M, Broxholme J, Kerr B, Salmon A, Ostman-Smith I, Watkins H. Mutations in the gamma(2) subunit of AMP-activated protein kinase cause familial hypertrophic cardiomyopathy: evidence for the central role of energy compromise in disease pathogenesis. *Hum Mol Genet*. 2001;**10**:1215-1220.

Böhm J, Yiş U, Ortaç R, Cakmakçı H, Kurul SH, Dirik E, Laporte J. Case report of intrafamilial variability in autosomal recessive centronuclear myopathy associated to a novel BIN1 stop mutation. *Orphanet J Rare Dis*. 2010;**5**:35.

Brauch, K.M., M.L. Karst, K.J. Herron, M. de Andrade, P.A. Pellikka, R.J. Rodeheffer, V.V. Michels, and T.M. Olson, Mutations in ribonucleic acid binding protein gene cause familial dilated cardiomyopathy. *J Am Coll Cardiol*. 2009;**54**:930-941.

Brugada R, Hong K, Dumaine R, Cordeiro J, Gaita F, Borggrefe M, Menendez TM, Brugada J, Pollevick GD, Wolpert C, Burashnikov E, Matsuo K, Wu YS, Guerchicoff A, Bianchi F, Giustetto C, Schimpf R, Brugada P, Antzelevitch C. Sudden death associated with short-QT syndrome linked to mutations in HERG. *Circulation*. 2004;**109**:30-35.

Brunetti-Pierri N, Scaglia F. GM1 gangliosidosis: review of clinical, molecular, and therapeutic aspects. *Mol Genet Metab*. 2008;**94**:391-396.

Chen J, Hong D, Wang Z, Yuan Y. A novel PNPLA2 mutation causes neutral lipid storage disease with myopathy (NLSDM) presenting muscular dystrophic features with lipid storage and rimmed vacuoles. *Clin Neuropathol*. 2010;**29**:351-356.

Chen L, Marquardt ML, Tester DJ, Sampson KJ, Ackerman MJ, Kass RS. Mutation of an A-kinase-anchoring protein causes long-QT syndrome. *Proc Natl Acad Sci U S A*. 2007;**104**:20990-20995.

Chen YH, Xu SJ, Bendahhou S, Wang XL, Wang Y, Xu WY, Jin HW, Sun H, Su XY, Zhuang QN, Yang YQ, Li YB, Liu Y, Xu HJ, Li XF, Ma N, Mou CP, Chen Z, Barhanin J, Huang W. KCNQ1 gain-of-function mutation in familial atrial fibrillation. *Science*. 2003;**299**:251-254.

Chiu C, Tebo M, Ingles J, Yeates L, Arthur JW, Lind JM, Semsarian C. Genetic screening of calcium regulation genes in familial hypertrophic cardiomyopathy. *J Mol Cell Cardiol*. 2007;**43**:337-343.

Cízková A, Stránecký V, Mayr JA, Tesarová M, Havlícková V, Paul J, Ivánek R, Kuss AW, Hansíková H, Kaplanová V, Vrbacký M, Hartmannová H, Nosková L, Honzík T, Drahota Z, Magner M, Hejzlarová K, Sperl W, Zeman J, Houstek J, Kmoch S. TMEM70 mutations cause isolated ATP synthase deficiency and neonatal mitochondrial encephalocardiomyopathy. *Nat Genet*. 2008;**40**:1288-1290.

Clayton PT, Winchester BG, Keir G. Hypertrophic obstructive cardiomyopathy in a neonate with the carbohydrate-deficient glycoprotein syndrome. J *Inherit Metab Dis*. 1992;**15**:857–861.

Collin GB, Marshall JD, Ikeda A, So WV, Russell-Eggitt I, Maffei P, Beck S, Boerkoel CF, Sicolo N, Martin M, Nishina PM, Naggert JK. Mutations in ALMS1 cause obesity, type 2 diabetes and neurosensory degeneration in Alström syndrome. *Nat Genet*. 2002;**31**:74-78.

Cutler DJ, Isner JM, Bracey AW, Hufnagel CA, Conrad PW, Roberts WC, Kerwin DM, Weintraub AM. Hemochromatosis heart disease: an unemphasized cause of potentially reversible restrictive cardiomyopathy. *Am J Med*. 1980;**69**:923-928.

Davey KM, Parboosingh JS, McLeod DR, Chan A, Casey R, Ferreira P, Snyder FF, Bridge PJ, Bernier FP. Mutation of DNAJC19, a human homologue of yeast inner mitochondrial membrane co-chaperones, causes DCMA syndrome, a novel autosomal recessive Barth syndrome-like condition. *J Med Genet*. 2006;**43**:385-393.

Davis JS, Hassanzadeh S, Winitsky S, Lin H, Satorius C, Vemuri R, Aletras AH, Wen H, Epstein ND. The overall pattern of cardiac contraction depends on a spatial gradient of myosin regulatory light chain phosphorylation. *Cell*. 2001;**107**:631-641.

Day JW, Ricker K, Jacobsen JF, Rasmussen LJ, Dick KA, Kress W, Schneider C, Koch MC, Beilman GJ, Harrison AR, Dalton JC, Ranum LP. Myotonic dystrophy type 2: molecular, diagnostic and clinical spectrum. *Neurology*. 2003;**60**:657-664.

De Gobbi M, Roetto A, Piperno A, Mariani R, Alberti F, Papanikolaou G, Politou , Lockitch G, Girelli D, Fargion S, Cox TM, Gasparini P, Cazzola M, Camaschella C. Natural history of juvenile haemochromatosis. *Br J Haematol*. 2002;**117**:973-979.

Delpón E, Cordeiro JM, Núñez L, Thomsen PE, Guerchicoff A, Pollevick GD, Wu Y, Kanters JK, Larsen CT, Hofman-Bang J, Burashnikov E, Christiansen M, Antzelevitch C. Functional effects of KCNE3 mutation and its role in the development of Brugada syndrome. *Circ Arrhythm Electrophysiol*. 2008;**1**:209-218.

den Boer ME, Wanders RJ, Morris AA, IJlst L, Heymans HS, Wijburg FA. Long-chain 3-hydroxyacyl-CoA dehydrogenase deficiency: clinical presentation and follow-up of 50 patients. *Pediatrics*. 2002;**109**:99-104.

Duboscq-Bidot L, Xu P, Charron P, Neyroud N, Dilanian G, Millaire A, Bors V, Komajda M, Villard E. Mutations in the Z-band protein myopalladin gene and idiopathic dilated cardiomyopathy. *Cardiovasc Res*. 2008;**77**:118-125.

Duncan AJ, Bitner-Glindzicz M, Meunier B, Costello H, Hargreaves IP, López LC, Hirano M, Quinzii CM, Sadowski MI, Hardy J, Singleton A, Clayton PT, Rahman S. A nonsense mutation in COQ9 causes autosomal-recessive neonatal-onset primary coenzyme Q10 deficiency: a potentially treatable form of mitochondrial disease. *Am J Hum Genet*. 2009;**84**:558-566.

Dürr A, Cossee M, Agid Y, Campuzano V, Mignard C, Penet C, Mandel JL, Brice A, Koenig M. Clinical and genetic abnormalities in patients with Friedreich's ataxia. *N Engl J Med*. 1996;**335**:1169-1175.

Eckhardt LL, Farley AL, Rodriguez E, Ruwaldt K, Hammill D, Tester DJ, Ackerman MJ, Makielski JC. KCNJ2 mutations in arrhythmia patients referred for LQT testing: a mutation T305A with novel effect on rectification properties. *Heart Rhythm*. **2007**;4:323-329.

Edwards MA, Green A, Colli A, Rylance G. Tyrosinaemia type I and hypertrophic obstructive cardiomyopathy. *Lancet*. 1987 1:1437-1438.

Elleder M, Bradová V, Smíd F, Budĕsínský M, Harzer K, Kustermann-Kuhn B, Ledvinová J, Bĕlohlávek, Král V, Dorazilová V. Cardiocyte storage and hypertrophy as a sole manifestation of Fabry's disease. Report on a case simulating hypertrophic non-obstructive cardiomyopathy. *Virchows Arch A Pathol Anat Histopathol*. 1990;**417**:449-455.

Erdmann J, Hassfeld S, Kallisch H, Fleck, E, Regitz-Zagrose, V. Genetic variants in the promoter (g983G>T) and coding region (A92T) of the human cardiotrophin-1 gene (CTF1) in patients with dilated cardiomyopathy. *Hum. Mutat*. 2000;**16**:448.

Fatkin D, MacRae C, Sasaki T, Wolff M, Porcu M, Frenneaux M, Atherton J, Vidaillet H, Spudich S, Girolami U, Seidman J, Seidman C. Missense mutations in the rod domain of the lamin A/C gene as causes of dilated cardiomyopathy and conduction-system disease. *N Engl J Med*. 1999;**341**:1715–1724.

FitzPatrick DR, Hill A, Tolmie JL, Thorburn DR, Christodoulou J. The molecular basis of malonyl-CoA decarboxylase deficiency. *Am J Hum Genet*. 1999;**65**:318-326.

Geisterfer-Lowrance AA, Kass S, Tanigawa G, Vosberg HP, McKenna W, Seidman CE, Seidman JG. A molecular basis for familial hypertrophic cardiomyopathy: a beta cardiac myosin heavy chain gene missense mutation. *Cell*. 1990;**62**:999-1006.

Gerull B, Gramlich M, Atherton J, McNabb M, Trombitás K, Sasse-Klaassen S, Seidman JG, Seidman C, Granzier H, Labeit S, Frenneaux M, Thierfelder L. Mutations of TTN, encoding the giant muscle filament titin, cause familial dilated cardiomyopathy. *Nat Genet*. 2002;**30**:201-204.

Gerull B, Heuser A, Wichter T, Paul M, Basson CT, McDermott DA, Lerman BB, Markowitz SM, Ellinor PT, MacRae CA, Peters S, Grossmann KS, Drenckhahn J, Michely B, Sasse-Klaassen S, Birchmeier W, Dietz R, Breithardt G, Schulze-Bahr E, Thierfelder L. Mutations in the desmosomal protein plakophilin-2 are common in arrhythmogenic right ventricular cardiomyopathy. *Nat Genet*. 2004;**36**:1162-1164.

Gollob MH, Green MS, Tang AS, Gollob T, Karibe A, Ali Hassan AS, Ahmad F, Lozado R, Shah G, Fananapazir L, Bachinski LL, Roberts R. Identification of a gene responsible for familial Wolff-Parkinson-White syndrome. *N Engl J Med*. 2001;**344**:1823-1831.

Gollob MH, Jones DL, Krahn AD, Danis L, Gong XQ, Shao Q, Liu X, Veinot JP, Tang AS, Stewart AF, Tesson F, Klein GJ, Yee R, Skanes AC, Guiraudon GM, Ebihara L, Bai D. Somatic mutations in the connexin 40 gene (GJA5) in atrial fibrillation. *N Engl J Med*. 2006;**354**:2677-2688.

Götz A, Tyynismaa H, Euro L, Ellonen P, Hyötyläinen T, Ojala T, Hämäläinen RH, Tommiska J, Raivio T, Oresic M, Karikoski R, Tammela O, Simola KO, Paetau A, Tyni T, Suomalainen A. Exome sequencing identifies mitochondrial alanyl-tRNA synthetase mutations in infantile mitochondrial cardiomyopathy. *Am J Hum Genet*. 2011;**88**:635-642.

Gripp KW, Lin AE, Nicholson L, Allen W, Cramer A, Jones KL, Kutz W, Peck D, Rebolledo MA, Wheeler PG, Wilson W, Al-Rahawan MM, Stabley DL, Sol-Church K. Further delineation of the phenotype resulting from BRAF or MEK1 germline mutations helps differentiate cardio-facio-cutaneous syndrome from Costello syndrome. *Am J Med Genet A*. 2007;**143A**:1472-1480.

Guertl B, Noehammer C, Hoefler G. Metabolic cardiomyopathies. *Int J Exp Pathol*. 2000;**81**:349-372.

Haïssaguerre M, Chatel S, Sacher F, Weerasooriya R, Probst V, Loussouarn G, Horlitz M, Liersch R, Schulze-Bahr E, Wilde A, Kääb S, Koster J, Rudy Y, Le Marec H, Schott JJ. Ventricular fibrillation with prominent early repolarization associated with a rare variant of KCNJ8/KATP channel. *J Cardiovasc Electrophysiol*. 2009;**20**:93-98.

Hamidi Asl L, Liepnieks JJ, Hamidi Asl K, Uemichi T, Moulin G, Desjoyaux E, Loire R, Delpech M, Grateau G, Benson MD. Hereditary amyloid cardiomyopathy caused by a variant apolipoprotein A1. *Am J Pathol*. 1999;**154**:221-227.

Hannuksela J, Leppilampi M, Peuhkurinen K, Kärkkäinen S, Saastamoinen E, Heliö T, Kaartinen M, Nieminen MS, Nieminen P, Parkkila S. Hereditary hemochromatosis gene (HFE) mutations C282Y, H63D and S65C in patients with idiopathic dilated cardiomyopathy. *Eur J Heart Fail*. 2005;**7**:103-108.

Hassel D, Dahme T, Erdmann J, Meder B, Huge A, Stoll M, Just S, Hess A, Ehlermann P, Weichenhan D, Grimmler M, Liptau H, Hetzer R, Regitz-Zagrosek V, Fischer C, Nürnberg P, Schunkert H, Katus HA, Rottbauer W. Nexilin mutations destabilize cardiac Z-disks and lead to dilated cardiomyopathy. *Nat Med*. 2009;**15**:1281-1288.

Hayashi T, Arimura T, Itoh-Satoh M, Ueda K, Hohda S, Inagaki N, Takahashi M, Hori H, Yasunami M, Nishi H, Koga Y, Nakamura H, Matsuzaki M, Choi BY, Bae SW, You CW, Han KH, Park JE, Knöll R, Hoshijima M, Chien KR, Kimura A. Tcap gene mutations in hypertrophic cardiomyopathy and dilated cardiomyopathy. *J Am Coll Cardiol*. 2004a;**44**:2192-2201.

Hayashi T, Arimura T, Ueda K, Shibata H, Hohda S, Takahashi M, Hori H, Koga Y, Oka N, Imaizumi T, Yasunami M, Kimura A. Identification and functional analysis of a caveolin-3 mutation associated with familial hypertrophic cardiomyopathy. *Biochem Biophys Res Commun*. 2004b;**313**:178-184.

He M, Rutledge SL, Kelly DR, Palmer CA, Murdoch G, Majumder N, Nicholls RD, Pei Z, Watkins PA, Vockley J. A new genetic disorder in mitochondrial fatty acid beta-oxidation: ACAD9 deficiency. *Am J Hum Genet*. 2007;**81**:87-103.

Heuser A, Plovie ER, Ellinor PT, Grossmann KS, Shin JT, Wichter T, Basson CT, Lerman BB, Sasse-Klaassen S, Thierfelder L, MacRae CA, Gerull B. Mutant desmocollin-2 causes arrhythmogenic right ventricular cardiomyopathy. *Am J Hum Genet*. 2006;**79**:1081-1088.

Hodgson-Zingman DM, Karst ML, Zingman LV, Heublein DM, Darbar D, Herron KJ, Ballew JD, de Andrade M, Burnett JC Jr, Olson TM. Atrial natriuretic peptide frameshift mutation in familial atrial fibrillation. *N Engl J Med*. 2008;**359**:158-165.

Hoefs SJ, Dieteren CE, Distelmaier F, Janssen RJ, Epplen A, Swarts HG, Forkink M, Rodenburg RJ, Nijtmans LG, Willems PH, Smeitink JA, van den Heuvel LP. NDUFA2 complex I mutation leads to Leigh disease. *Am J Hum Genet*. 2008;**82**:1306-1315.

Hoffmann B, Schmidt-Traub H, Perrot A, Osterziel KJ, Gessner R. First mutation in cardiac troponin C, L29Q, in a patient with hypertrophic cardiomyopathy. *Hum Mutat*. 2001;**17**:524.

Holm H, Gudbjartsson DF, Sulem P, Masson G, Helgadottir HT, Zanon C, Magnusson OT, Helgason A, Saemundsdottir J, Gylfason A, Stefansdottir H, Gretarsdottir S, Matthiasson SE, Thorgeirsson GM, Jonasdottir A, Sigurdsson A, Stefansson H, WergeT, Rafnar T, Kiemeney LA, Parvez B, Muhammad R, Roden DM, Darbar D, Thorleifsson G, Walters GB, Kong A, Thorsteinsdottir U, Arnar DO, Stefansson K. A rare variant in MYH6 is associated with high risk of sick sinus syndrome. *Nat Genet*. 2011;**43**:316-320.

Hu D, Barajas-Martinez H, Burashnikov E, Springer M, Wu Y, Varro A, Pfeiffer R, Koopmann TT, Cordeiro JM, Guerchicoff A, Pollevick GD, Antzelevitch C. A mutation in the beta 3 subunit of the cardiac sodium channel associated with Brugada ECG phenotype. *Circ Cardiovasc Genet*. 2009;**2**:270-278.

Hübbers CU, Clemen CS, Kesper K, Böddrich A, Hofmann A, Kämäräinen O, Tolksdorf K, Stumpf M, Reichelt J, Roth U, Krause S, Watts G, Kimonis V, Wattjes MP, Reimann J, Thal DR, Biermann K, Evert BO, Lochmüller H, Wanker EE, Schoser BG, Noegel AA, Schröder R. Pathological consequences of VCP mutations on human striated muscle. *Brain* 2007;**130**:381-393.

Huigsloot M, Nijtmans LG, Szklarczyk R, Baars MJ, van den Brand MA, Hendriksfranssen MG, van den Heuvel LP, Smeitink JA, Huynen MA, Rodenburg RJ. A mutation in C2orf64 causes impaired cytochrome c oxidase assembly and mitochondrial cardiomyopathy. *Am J Hum Genet*. 2011;**88**:488-493.

Ichida F, Tsubata S, Bowles KR, Haneda N, Uese K, Miyawaki T, Dreyer WJ, Messina J, Li H, Bowles NE, Towbin JA. Novel gene mutations in patients with left ventricular noncompaction or Barth syndrome. *Circulation*. 2001;**103**:1256-1263.

Kattygnarath D, Maugenre S, Neyroud N, Balse E, Ichai C, Denjoy I, Dilanian G, Martins RP, Fressart V, Berthet M, Schott JJ, Leenhardt A, Probst V, Le Marec H, Hainque B, Coulombe A, Hatem SN, Guicheney P. MOG1: a new susceptibility gene for Brugada syndrome. *Circ Cardiovasc Genet*. 2011;**4**:261-268.

Kimura A, Harada H, Park JE, Nishi H, Satoh M, Takahashi M, Hiroi S, Sasaoka T, Ohbuchi N, Nakamura T, Koyanagi T, Hwang TH, Choo JA, Chung KS, Hasegawa A, Nagai R, Okazaki O, Nakamura H, Matsuzaki M, Sakamoto T, Toshima H, Koga Y, Imaizumi T, Sasazuki T. Mutations in the cardiac troponin I gene associated with hypertrophic cardiomyopathy. *Nat Genet*. 1997;**16**:379-382.

Kirk EP, Sunde M, Costa MW, Rankin SA, Wolstein O, Castro ML, Butler TL, Hyun C, Guo G, Otway R, Mackay JP, Waddell LB, Cole AD, Hayward C, Keogh A, Macdonald P, Griffiths L, Fatkin D, Sholler GF, Zorn AM, Feneley MP, Winlaw DS, Harvey RP. Mutations in cardiac T-box factor gene TBX20 are associated with diverse cardiac pathologies, including defects of septation and valvulogenesis and cardiomyopathy. *Am J Hum Genet*. 2007;**81**:280-291.

Knöll R, Hoshijima M, Hoffman HM, Person V, Lorenzen-Schmidt I, Bang ML, Hayashi T, Shiga N, Yasukawa H, Schaper W, McKenna W, Yokoyama M, Schork NJ, Omens JH, McCulloch AD, Kimura A, Gregorio CC, Poller W, Schaper J, Schultheiss HP, Chien KR. The cardiac mechanical stretch sensor machinery involves a Z disc complex that is defective in a subset of human dilated cardiomyopathy. *Cell*. 2002;**111**:943-955.

Knöll R, Postel R, Wang J, Krätzner R, Hennecke G, Vacaru AM, Vakeel P, Schubert C, Murthy K, Rana BK, Kube D, Knöll G, Schäfer K, Hayashi T, Holm T, Kimura A, Schork N, Toliat MR, Nürnberg P, Schultheiss HP, Schaper W, Schaper J, Bos E, Den Hertog J, van Eeden FJ, Peters PJ, Hasenfuss G, Chien KR, Bakkers J. Laminin-alpha4 and integrin-linked kinase mutations cause human cardiomyopathy via simultaneous defects in cardiomyocytes and endothelial cells. *Circulation*. 2007;**116**:515-525.

Koenig M, Hoffman EP, Bertelson CJ, Monaco AP, Feener C, Kunkel LM. Complete cloning of the Duchenne muscular dystrophy (DMD) cDNA and preliminary genomic organization of the DMD gene in normal and affected individuals. *Cell*. 1987;**50**:509-517.

Kollberg G, Tulinius M, Gilljam T, Ostman-Smith I, Forsander G, Jotorp P, Oldfors A, Holme E. Cardiomyopathy and exercise intolerance in muscle glycogen storage disease 0. *N Engl J Med*. 2007;**357**:1507-1514.

Kruse M, Schulze-Bahr E, Corfield V, Beckmann A, Stallmeyer B, Kurtbay G, Ohmert I, Schulze-Bahr E, Brink P, Pongs O. Impaired endocytosis of the ion channel TRPM4 is associated with human progressive familial heart block type I. *J Clin Invest*. 2009;**119**:2737-2744.

Lahat H, Pras E, Olender T, Avidan N, Ben-Asher E, Man O, Levy-Nissenbaum E, Khoury A, Lorber A, Goldman B, Lancet D, Eldar M. A missense mutation in a highly conserved region of CASQ2 is associated with autosomal recessive catecholamine-induced polymorphic ventricular tachycardia in Bedouin families from Israel. *Am J Hum Genet*. 2001;**69**:1378-1384.

Landstrom AP, Weisleder N, Batalden KB, Bos JM, Tester DJ, Ommen SR, Wehrens XH, Claycomb WC, Ko JK, Hwang M, Pan Z, Ma J, Ackerman MJ. Mutations in JPH2-encoded junctophilin-2 associated with hypertrophic cardiomyopathy in humans. *J Mol Cell Cardiol*. 2007;**42**:1026-1035.

Lerman BB, Dong B, Stein KM, Markowitz SM, Linden J, Catanzaro DF. Right ventricular outflow tract tachycardia due to a somatic cell mutation in G protein subunitalphai2. *J Clin Invest*. 1998;**101**:2862-2868.

Levitas A, Muhammad E, Harel G, Saada A, Caspi VC, Manor E, Beck JC, Sheffield V, Parvari R. Familial neonatal isolated cardiomyopathy caused by a mutation in the flavoprotein subunit of succinate dehydrogenase. *Eur J Hum Genet*. 2010;**18**:1160-1165.

Li D, Parks SB, Kushner JD, Nauman D, Burgess D, Ludwigsen S, Partain J, Nixon RR, Allen CN, Irwin RP, Jakobs PM, Litt M, Hershberger RE. Mutations of presenilin genes in dilated cardiomyopathy and heart failure. *Am J Hum Genet*. 2006;**79**:1030-1039.

Li D, Tapscoft T, Gonzalez O, Burch PE, Quiñones MA, Zoghbi WA, Hill R, Bachinski LL, Mann DL, Roberts R. Desmin mutation responsible for idiopathic dilated cardiomyopathy. *Circulation*. 1999;**100**:461-464.

Limongelli G, Russo S, Digilio MC, Masciadri M, Pacileo G, Fratta F, Martone F, Maddaloni V, D'Alessandro R, Calabro P, Russo MG, Calabro R, Larizza L. Hypertrophic cardiomyopathy in a girl with Cornelia de Lange syndrome due to mutation in SMC1A. *Am J Med Genet* A. 2010;**152A**:2127-2129.

Limongelli G, Sarkozy A, Pacileo G, Calabrò P, Digilio MC, Maddaloni V, Gagliardi G, Di Salvo G, Iacomino M, Marino B, Dallapiccola B, Calabrò R. Genotype-phenotype analysis and natural history of left ventricular hypertrophy in LEOPARD syndrome. *Am J Med Genet A*. 2008;**146A**:620-628.

Loeffen J, Elpeleg O, Smeitink J, Smeets R, Stöckler-Ipsiroglu S, Mandel H, Sengers R, Trijbels F, van den Heuvel L. Mutations in the complex I NDUFS2 gene of patients with cardiomyopathy and encephalomyopathy. *Ann Neurol*. 2001;**49**:195-201.

Loeffen J, Smeitink J, Triepels R, Smeets R, Schuelke M, Sengers R, Trijbels F, Hamel B, Mullaart R, van den Heuvel L. The first nuclear-encoded complex I mutation in a patient with Leigh syndrome. *Am J Hum Genet*. 1998;**63**:1598-1608.

London B, Michalec M, Mehdi H, Zhu X, Kerchner L, Sanyal S, Viswanathan PC, Pfahnl AE, Shang LL, Madhusudanan M, Baty CJ, Lagana S, Aleong R, Gutmann R, Ackerman MJ, McNamara DM, Weiss R, Dudley SC Jr. Mutation in glycerol-3-phosphate dehydrogenase 1 like gene (GPD1-L) decreases cardiac Na+ current and causes inherited arrhythmias. *Circulation*. 2007;**116**:2260-2268.

Martinez HR, Belmont JW, Craigen WJ, Taylor MD, Jefferies JL. Left ventricular noncompaction in Sotos syndrome. *Am J Med Genet* A. 2011;**155A**:1115-1118.

Massin MM, Radermecker MA, Verloes A, Jacquot S, Grenade T. Cardiac involvement in Cofﬁn–Lowry syndrome. *Acta Paediatr*. 1999;**88**:468–470.

Mathur A, Sims HF, Gopalakrishnan D, Gibson B, Rinaldo P, Vockley J, Hug G, Strauss AW. Molecular heterogeneity in very-long-chain acyl-CoA dehydrogenase deficiency causing pediatric cardiomyopathy and sudden death. *Circulation*. 1999;**99**:1337-1343.

Mayr JA, Merkel O, Kohlwein SD, Gebhardt BR, Böhles H, Fötschl U, Koch J, Jaksch M, Lochmüller H, Horváth R, Freisinger P, Sperl W. Mitochondrial phosphate-carrier deficiency: a novel disorder of oxidative phosphorylation. *Am J Hum Genet*. 2007;**80**:478-484.

McKoy G, Protonotarios N, Crosby A, Tsatsopoulou A, Anastasakis A, Coonar A, Norman M, Baboonian C, Jeffery S, McKenna WJ. Identification of a deletion in plakoglobin in arrhythmogenic right ventricular cardiomyopathy with palmoplantar keratoderma and woolly hair (Naxos disease). *Lancet*. 2000;**355**:2119-2124.

McNair WP, Ku L, Taylor MR, Fain PR, Dao D, Wolfel E, Mestroni L; Familial Cardiomyopathy Registry Research Group. SCN5A mutation associated with dilated cardiomyopathy, conduction disorder, and arrhythmia. *Circulation*. 2004;**110**:2163-2167.

Medeiros-Domingo A, Kaku T, Tester DJ, Iturralde-Torres P, Itty A, Ye B, Valdivia C, Ueda K, Canizales-Quinteros S, Tusié-Luna MT, Makielski JC, Ackerman MJ. SCN4B-encoded sodium channel beta4 subunit in congenital long-QT syndrome. *Circulation*. 2007;**116**:134-142.

Medeiros-Domingo A, Tan BH, Crotti L, Tester DJ, Eckhardt L, Cuoretti A, Kroboth SL, Song C, Zhou Q, Kopp D, Schwartz PJ, Makielski JC, Ackerman MJ. Gain-of-function mutation S422L in the KCNJ8-encoded cardiac K(ATP) channel Kir6.1 as a pathogenic substrate for J-wave syndromes. *Heart Rhythm*. 2010;**7**:1466-1471.

Merner ND, Hodgkinson KA, Haywood AFM, Connors S, French VM, Drenckhahn JD, Kupprion C, Ramadanova K, Thierfelder L, McKenna W, Gallagher B, Morris-Larkin L, Bassett AS, Parfrey PS, Young TL. Arrhythmogenic right ventricular cardiomyopathy type 5 is a fully penetrant, lethal disorder caused by a missense mutation in the TMEM43 gene. *Am J Hum Genet*. 2008;**82**:809-821.

Milanesi R, Baruscotti M, Gnecchi-Ruscone T, DiFrancesco D. Familial sinus bradycardia associated with a mutation in the cardiac pacemaker channel. *N Engl J Med*. 2006;**354**:151-157.

Miskinyte S, Butler MG, Hervé D, Sarret C, Nicolino M, Petralia JD, Bergametti F, Arnould M, Pham VN, Gore AV, Spengos K, Gazal S, Woimant F, Steinberg GK, Weinstein BM, Tournier-Lasserve E. Loss of BRCC3 deubiquitinating enzyme leads to abnormal angiogenesis and is associated with syndromic moyamoya. *Am J Hum Genet*. 2011;**88**:718-728.

Mohapatra B, Jimenez S, Lin JH, Bowles KR, Coveler KJ, Marx JG, Chrisco MA, Murphy RT, Lurie PR, Schwartz RJ, Elliott PM, Vatta M, McKenna W, Towbin JA, Bowles NE. Mutations in the muscle LIM protein and alpha-actinin-2 genes in dilated cardiomyopathy and endocardial fibroelastosis. *Mol Genet Metab*. 2003;**80**:207-215.

Mohiddin SA, Ahmed ZM, Griffith AJ, Tripodi D, Friedman TB, Fananapazir L, Morell RJ. Novel association of hypertrophic cardiomyopathy, sensorineural deafness, and a mutation in unconventional myosin VI (MYO6). *J Med Genet*. 2004;**41**:309-314.

Mohler PJ, Schott JJ, Gramolini AO, Dilly KW, Guatimosim S, duBell WH, Song LS, Haurogné K, Kyndt F, Ali ME, Rogers TB, Lederer WJ, Escande D, Le Marec H, Bennett V. Ankyrin-B mutation causes type 4 long-QT cardiac arrhythmia and sudden cardiac death. *Nature*. 2003;**421**:634-639.

Monserrat L, Gimeno-Blanes JR, Marín F, Hermida-Prieto M, García-Honrubia A, Pérez I, Fernández X, de Nicolas R, de la Morena G, Payá E, Yagüe J, Egido J. Prevalence of fabry disease in a cohort of 508 unrelated patients with hypertrophic cardiomyopathy. *J Am Coll Cardiol*. 2007;**50**:2399-2403.

Mörner S, Hellman U, Suhr OB, Kazzam E, Waldenström A. Amyloid heart disease mimicking hypertrophic cardiomyopathy. *J Intern Med*. 2005;**258**:225-230.

Moslemi AR, Lindberg C, Nilsson J, Tajsharghi H, Andersson B, Oldfors A. Glycogenin-1 deficiency and inactivated priming of glycogen synthesis. *N Engl J Med*. 2010;**362**:1203-1210.

Mourad G, Delabre JP, Garrigue V. Cardiac amyloidosis with the E526V mutation of the fibrinogen A alpha-chain. *N Engl J Med*. 2008;**359**:2847-2848.

Müller T, Krasnianski M, Witthaut R, Deschauer M, Zierz S. Dilated cardiomyopathy may be an early sign of the C826A Fukutin-related protein mutation. *Neuromuscul Disord*. 2005;**15**:372-376.

Murakami T, Hayashi YK, Noguchi S, Ogawa M, Nonaka I, Tanabe Y, Ogino M, Takada F, Eriguchi M, Kotooka N, Campbell KP, Osawa M, Nishino I. Fukutin gene mutations cause dilated cardiomyopathy with minimal muscle weakness. *Ann Neurol*. 2006;**60**:597-602.

Narumi Y, Aoki Y, Niihori T, Neri G, Cavé H, Verloes A, Nava C, Kavamura MI, Okamoto N, Kurosawa K, Hennekam RC, Wilson LC, Gillessen-Kaesbach G, Wieczorek D, Lapunzina P, Ohashi H, Makita Y, Kondo I, Tsuchiya S, Ito E, Sameshima K, Kato K, Kure S, Matsubara Y. Molecular and clinical characterization of cardio-facio-cutaneous (CFC) syndrome: overlapping clinical manifestations with Costello syndrome. *Am J Med Genet* A. 2007;**143A**:799-807.

Nezu J, Tamai I, Oku A, Ohashi R, Yabuuchi H, Hashimoto N, Nikaido H, Sai Y, Koizumi A, Shoji Y, Takada G, Matsuishi T, Yoshino M, Kato H, Ohura T, Tsujimoto G, Hayakawa J, Shimane M, Tsuji A. Primary systemic carnitine deficiency is caused by mutations in a gene encoding sodium ion-dependent carnitine transporter. *Nat Genet*. **1999**;21:91-94.

Niihori T, Aoki Y, Narumi Y, Neri G, Cavé H, Verloes A, Okamoto N, Hennekam RC, Gillessen-Kaesbach G, Wieczorek D, Kavamura MI, Kurosawa K, Ohashi H, Wilson L, Heron D, Bonneau D, Corona G, Kaname T, Naritomi K, Baumann C, Matsumoto N, Kato K, Kure S, Matsubara Y. Germline KRAS and BRAF mutations in cardio-facio-cutaneous syndrome. *Nat Genet*. 2006;**38**:294-296.

Nishino I, Fu J, Tanji K, Yamada T, Shimojo S, Koori T, Mora M, Riggs JE, Oh SJ, Koga Y, Sue CM, Yamamoto A, Murakami N, Shanske S, Byrne E, Bonilla E, Nonaka I, DiMauro S, Hirano M. Primary LAMP-2 deficiency causes X-linked vacuolar cardiomyopathy and myopathy (Danon disease). *Nature*. 2000;**406**:906-910.

Ohno S, Zankov DP, Yoshida H, Tsuji K, Makiyama T, Itoh H, Akao M, Hancox JC, Kita T, Horie M. N- and C-terminal KCNE1 mutations cause distinct phenotypes of long QT syndrome. *Heart Rhythm*. 2007;**4**:332-340.

Olson TM, Alekseev AE, Liu XK, Park S, Zingman LV, Bienengraeber M, Sattiraju S, Ballew JD, Jahangir A, Terzic A. Kv1.5 channelopathy due to KCNA5 loss-of-function mutation causes human atrial fibrillation. *Hum Mol Genet*. 2006;**15**:2185-2191.

Olson TM, Illenberger S, Kishimoto NY, Huttelmaier S, Keating MT, Jockusch BM. Metavinculin mutations alter actin interaction in dilated cardiomyopathy. *Circulation* 2002;**105**:431-437.

Olson TM, Michels VV, Thibodeau SN, Tai YS, Keating MT. Actin mutations in dilated cardiomyopathy, a heritable form of heart failure. *Science*. 1998;**280**:750-752.

Osio A, Tan L, Chen SN, Lombardi R, Nagueh SF, Shete S, Roberts R, Willerson JT, Marian AJ. Myozenin 2 is a novel gene for human hypertrophic cardiomyopathy. *Circ Res*. 2007;**100**:766-768.

Ouyang P, Saarel E, Bai Y, Luo C, Lv Q, Xu Y, Wang F, Fan C, Younoszai A, Chen Q, Tu X, Wang QK. A de novo mutation in NKX2.5 associated with atrial septal defects, ventricular noncompaction, syncope and sudden death. *Clin Chim Acta*. 2011;**412**:170-175.

Palmieri L, Alberio S, Pisano I, Lodi T, Meznaric-Petrusa M, Zidar J, Santoro A, Scarcia P, Fontanesi F, Lamantea E, Ferrero I, Zeviani M. Complete loss-of-function of the heart/muscle-specific adenine nucleotide translocator is associated with mitochondrial myopathy and cardiomyopathy. *Hum Mol Genet*. 2005;**14**:3079-3088.

Pandit B, Sarkozy A, Pennacchio LA, Carta C, Oishi K, Martinelli S, Pogna EA, Schackwitz W, Ustaszewska A, Landstrom A, Bos JM, Ommen SR, Esposito G, Lepri F, Faul C, Mundel P, López Siguero JP, Tenconi R, Selicorni A, Rossi C, Mazzanti L, Torrente I, Marino B, Digilio MC, Zampino G, Ackerman MJ, Dallapiccola B, Tartaglia M, Gelb BD. Gain-of-function RAF1 mutations cause Noonan and LEOPARD syndromes with hypertrophic cardiomyopathy. *Nat Genet*. 2007;**39**:1007-1012.

Papadopoulou LC, Sue CM, Davidson MM, Tanji K, Nishino I, Sadlock JE, Krishna S, Walker W, Selby J, Glerum DM, Coster RV, Lyon G, Scalais E, Lebel R, Kaplan P, Shanske S, De Vivo DC, Bonilla E, Hirano M, DiMauro S, Schon EA. Fatal infantile cardioencephalomyopathy with COX deficiency and mutations in SCO2, a COX assembly gene. *Nat Genet*. 1999;**23**:333-337.

Papanikolaou G, Samuels ME, Ludwig EH, MacDonald ML, Franchini PL, Dubé MP, Andres L, MacFarlane J, Sakellaropoulos N, Politou M, Nemeth E, Thompson J, Risler JK, Zaborowska C, Babakaiff R, Radomski CC, Pape TD, Davidas O, Christakis J, Brissot P, Lockitch G, Ganz T, Hayden MR, Goldberg YP. Mutations in HFE2 cause iron overload in chromosome 1q-linked juvenile hemochromatosis. *Nat Genet*. 2004;**36**:77-82.

Phillips MF, Harper PS. Cardiac disease in myotonic dystrophy. *Cardiovasc Res*. 1997;**33**:13-22.

Pilichou K, Nava A, Basso C, Beffagna G, Bauce B, Lorenzon A, Frigo G, Vettori A, Valente M, Towbin J, Thiene G, Danieli GA, Rampazzo A. Mutations in desmoglein-2 gene are associated with arrhythmogenic right ventricular cardiomyopathy. *Circulation*. 2006;**113**:1171-1179.

Poetter K, Jiang H, Hassanzadeh S, Master SR, Chang A, Dalakas MC, Rayment I, Sellers JR, Fananapazir L, Epstein ND. Mutations in either the essential or regulatory light chains of myosin are associated with a rare myopathy in human heart and skeletal muscle. *Nat Genet*. 1996;**13**:63-69.

Pompe JC. Over idiopathische hypertrophie van het hart. *Ned Tijdschr Geneeskd*. 1931;**76**:304–311.

Posch MG, Boldt LH, Polotzki M, Richter S, Rolf S, Perrot A, Dietz R, Ozcelik C, Haverkamp W. Mutations in the cardiac transcription factor GATA4 in patients with lone atrial fibrillation. *Eur J Med Genet*. 2010;**53**:201-203.

Purevjav E, Varela J, Morgado M, Kearney DL, Li H, Taylor MD, Arimura T, Moncman CL, McKenna W, Murphy RT, Labeit S, Vatta M, Bowles NE, Kimura A, Boriek AM, Towbin JA. Nebulette mutations are associated with dilated cardiomyopathy and endocardial fibroelastosis. *J Am Coll Cardiol*. 2010;**56**:1493-1502.

Rajab A, Straub V, McCann LJ, Seelow D, Varon R, Barresi R, Schulze A, Lucke B, Lützkendorf S, Karbasiyan M, Bachmann S, Spuler S, Schuelke M. Fatal cardiac arrhythmia and long-QT syndrome in a new form of congenital generalized lipodystrophy with muscle rippling (CGL4) due to PTRF-CAVIN mutations. *PLoS Genet*. 2010;**6**:e1000874.

Rampazzo A, Nava A, Malacrida S, Beffagna G, Bauce B, Rossi V, Zimbello R, Simionati B, Basso C, Thiene G, Towbin JA, Danieli GA. Mutation in human desmoplakin domain binding to plakoglobin causes a dominant form of arrhythmogenic right ventricular cardiomyopathy. *Am J Hum Genet*. 2002;**71**:1200-1206.

Roberts AE, Araki T, Swanson KD, Montgomery KT, Schiripo TA, Joshi VA, Li L, Yassin Y, Tamburino AM, Neel BG, Kucherlapati RS. Germline gain-of-function mutations in SOS1 cause Noonan syndrome. *Nat Genet*. 2007;**39**:70-74.

Roetto A, Papanikolaou G, Politou M, Alberti F, Girelli D, Christakis J, Loukopoulos D, Camaschella C. Mutant antimicrobial peptide hepcidin is associated with severe juvenile hemochromatosis. *Nat Genet*. 2003;**33**:21-22.

Rope AF, Wang K, Evjenth R, Xing J, Johnston JJ, Swensen JJ, Johnson WE, Moore B, Huff CD, Bird LM, Carey JC, Opitz JM, Stevens CA, Jiang T, Schank C, Fain HD, Robison R, Dalley B, Chin S, South ST, Pysher TJ, Jorde LB, Hakonarson H, Lillehaug JR, Biesecker LG, Yandell M, Arnesen T, Lyon GJ. Using VAAST to identify an X-linked disorder resulting in lethality in male infants due to N-terminal acetyltransferase deficiency. *Am J Hum Genet*. 2011;**89**:28-43.

Saada A, Shaag A, Arnon S, Dolfin T, Miller C, Fuchs-Telem D, Lombes A, Elpeleg O. Antenatal mitochondrial disease caused by mitochondrial ribosomal protein (MRPS22) mutation. *J Med Genet*. 2007;**44**:784-786.

Sakata K, Shimizu M, Ino H, Yamaguchi M, Terai H, Fujino N, Hayashi K, Kaneda T, Inoue M, Oda Y, Fujita T, Kaku B, Kanaya H, Mabuchi H. High incidence of sudden cardiac death with conduction disturbances and atrial cardiomyopathy caused by a nonsense mutation in the STA gene. *Circulation*. 2005;**111**:3352-3358.

Saraiva MJ. Transthyretin mutations in health and disease. *Hum Mutat*. 1995;**5**:191-196.

Satoh M, Takahashi M, Sakamoto T, Hiroe M, Marumo F, Kimura A. Structural analysis of the titin gene in hypertrophic cardiomyopathy: identification of a novel disease gene. *Biochem Biophys Res Commun*. 1999;**262**:411-417.

Schmitt JP, Kamisago M, Asahi M, Li GH, Ahmad F, Mende U, Kranias EG, MacLennan DH, Seidman JG, Seidman CE. Dilated cardiomyopathy and heart failure caused by a mutation in phospholamban. *Science* 2003;**299**:1410 -1413.

Schneider-Gold C, Beer M, Köstler H, Buchner S, Sandstede J, Hahn D, Toyka KV. Cardiac and skeletal muscle involvement in myotonic dystrophy type 2 (DM2): a quantitative 31P-MRS and MRI study. *Muscle Nerve*. 2004;**30**:636-644.

Schönberger J, Wang L, Shin JT, Kim SD, Depreux FF, Zhu H, Zon L, Pizard A, Kim JB, Macrae CA, Mungall AJ, Seidman JG, Seidman CE. Mutation in the transcriptional coactivator EYA4 causes dilated cardiomyopathy and sensorineural hearing loss. *Nat Genet*. 2005;**37**:418-422.

Schott JJ, Benson DW, Basson CT, Pease W, Silberbach GM, Moak JP, Maron BJ, Seidman CE, Seidman JG. Congenital heart disease caused by mutations in the transcription factor NKX2.5. *Science* 1998;**281**:108-111.

Schubbert S, Zenker M, Rowe SL, Böll S, Klein C, Bollag G, van der Burgt I, Musante L, Kalscheuer V, Wehner LE, Nguyen H, West B, Zhang KY, Sistermans E, Rauch A, Niemeyer CM, Shannon K, Kratz CP. Germline KRAS mutations cause Noonan syndrome. *Nat Genet*. 2006;**38**:331-336.

Schulze-Bahr E, Neu A, Friederich P, Kaupp UB, Breithardt G, Pongs O, Isbrandt D. Pacemaker channel dysfunction in a patient with sinus node disease. *J Clin Invest*. 2003;**111**:1537-1545.

Selcen D, Engel AG. Mutations in myotilin cause myofibrillar myopathy. *Neurology*. 2004;**62**:1363-1371.

Selcen D, Muntoni F, Burton BK, Pegoraro E, Sewry C, Bite AV, Engel AG. Mutation in BAG3 causes severe dominant childhood muscular dystrophy. *Ann Neurol*. 2009;**65**:83-89.

Sewell AC, Pontz BF, Weitzel D, Humburg C. Clinical heterogeneity in infantile galactosialidosis. *Eur J Pediatr*. 1987;**146**:528-531.

Shen J, Bao Y, Liu HM, Lee P, Leonard JV, Chen YT. Mutations in exon 3 of the glycogen debranching enzyme gene are associated with glycogen storage disease type III that is differentially expressed in liver and muscle. *J Clin Invest*. 1996;**98**:352-357.

Smeitink JA, Elpeleg O, Antonicka H, Diepstra H, Saada A, Smits P, Sasarman F, Vriend G, Jacob-Hirsch J, Shaag A, Rechavi G, Welling B, Horst J, Rodenburg RJ, van den Heuvel B, Shoubridge EA. Distinct clinical phenotypes associated with a mutation in the mitochondrial translation elongation factor EFTs. *Am J Hum Genet*. 2006;**79**:869-877.

Sovari AA, Bodine CK, Farokhi F. Cardiovascular manifestations of myotonic dystrophy-1. *Cardiol Rev*. 2007;**15**:191-194.

Sparkes R, Patton D, Bernier F. Cardiac features of a novel autosomal recessive dilated cardiomyopathic syndrome due to defective importation of mitochondrial protein. *Cardiol Young*. 2007;**17**:215-217.

Spiekerkoetter U, Sun B, Khuchua Z, Bennett MJ, Strauss AW. Molecular and phenotypic heterogeneity in mitochondrial trifunctional protein deficiency due to beta-subunit mutations. *Hum Mutat*. 2003;**21**:598-607.

Splawski I, Timothy KW, Sharpe LM, Decher N, Kumar P, Bloise R, Napolitano C, Schwartz PJ, Joseph RM, Condouris K, Tager-Flusberg H, Priori SG, Sanguinetti MC, Keating MT. Ca(V)1.2 calcium channel dysfunction causes a multisystem disorder including arrhythmia and autism. *Cell*. 2004;**119**:19-31.

Suzuki Y, Tsuji A, Omura K, Nakamura G, Awa S, Kroos M, Reuser AJ. Km mutant of acid alpha-glucosidase in a case of cardiomyopathy without signs of skeletal muscle involvement. *Clin Genet*. 1988;**33**:376-385.

Syrris P, Ward D, Evans A, Asimaki A, Gandjbakhch E, Sen-Chowdhry S, McKenna WJ. Arrhythmogenic right ventricular dysplasia/cardiomyopathy associated with mutations in the desmosomal gene desmocollin-2. *Am J Hum Genet*. 2006;**79**:978-984.

Tanigawa G, Jarcho JA, Kass S, Solomon SD, Vosberg HP, Seidman JG, Seidman CE. A molecular basis for familial hypertrophic cardiomyopathy: an alpha/beta cardiac myosin heavy chain hybrid gene. *Cell*. 1990;**62**:991-998.

Tartaglia M, Kalidas K, Shaw A, Song X, Musat DL, van der Burgt I, Brunner HG, Bertola DR, Crosby A, Ion A, Kucherlapati RS, Jeffery S, Patton MA, Gelb BD. PTPN11 mutations in Noonan syndrome: molecular spectrum, genotype-phenotype correlation, and phenotypic heterogeneity. *Am J Hum Genet*. 2002;**70**:1555-1563.

Tartaglia M, Pennacchio LA, Zhao C, Yadav KK, Fodale V, Sarkozy A, Pandit B, Oishi K, Martinelli S, Schackwitz W, Ustaszewska A, Martin J, Bristow J, Carta C, Lepri F, Neri C, Vasta I, Gibson K, Curry CJ, Siguero JP, Digilio MC, Zampino G, Dallapiccola B, Bar-Sagi D, Gelb BD. Gain-of-function SOS1 mutations cause a distinctive form of Noonan syndrome. *Nat Genet*. 2007;**39**:75-79.

Taylor MR, Slavov D, Gajewski A, Vlcek S, Ku L, Fain PR, Carniel E, Di Lenarda A, Sinagra G, Boucek MM, Cavanaugh J, Graw SL, Ruegg P, Feiger J, Zhu X, Ferguson DA, Bristow MR, Gotzmann J, Foisner R, Mestroni L; Familial Cardiomyopathy Registry Research Group. Thymopoietin (lamina-associated polypeptide 2) gene mutation associated with dilated cardiomyopathy. *Hum Mutat*. 2005;**26**:566-574.

Tein I, Elpeleg O, Ben-Zeev B, Korman SH, Lossos A, Lev D, Lerman-Sagie T, Leshinsky-Silver E, Vockley J, Berry GT, Lamhonwah AM, Matern D, Roe CR, Gregersen N. Short-chain acyl-CoA dehydrogenase gene mutation (c.319C>T) presents with clinical heterogeneity and is candidate founder mutation in individuals of Ashkenazi Jewish origin. *Mol Genet Metab*. 2008;**93**:179-189.

Thibodeau IL, Xu J, Li Q, Liu G, Lam K, Veinot JP, Birnie DH, Jones DL, Krahn AD, Lemery R, Nicholson BJ, Gollob MH. Paradigm of genetic mosaicism and lone atrial fibrillation: physiological characterization of a connexin 43-deletion mutant identified from atrial tissue. *Circulation*. 2010;**122**:236-244.

Thierfelder L, Watkins H, MacRae C, Lamas R, McKenna W, Vosberg HP, Seidman JG, Seidman CE. Alpha-tropomyosin and cardiac troponin T mutations cause familial hypertrophic cardiomyopathy: a disease of the sarcomere. *Cell*. 1994;**77**:701-712.

Thuillier L, Rostane H, Droin V, Demaugre F, Brivet M, Kadhom N, Prip-Buus C, Gobin S, Saudubray JM, Bonnefont JP. Correlation between genotype, metabolic data, and clinical presentation in carnitine palmitoyltransferase 2 (CPT2) deficiency. *Hum Mutat*. 2003;**21**:493-501.

Tiso N, Stephan DA, Nava A, Bagattin A, Devaney JM, Stanchi F, Larderet G, Brahmbhatt B, Brown K, Bauce B, Muriago M, Basso C, Thiene G, Danieli GA, Rampazzo A. Identification of mutations in the cardiac ryanodine receptor gene in families affected with arrhythmogenic right ventricular cardiomyopathy type 2 (ARVD2). *Hum Mol Genet*. 2001;**10**:189-194.

Tsubata S, Bowles KR, Vatta M, Zintz C, Titus J, Muhonen L, Bowles NE, Towbin JA. Mutations in the human delta-sarcoglycan gene in familial and sporadic dilated cardiomyopathy. *J Clin Invest*. 2000;**106**:655-662.

Ueda K, Valdivia C, Medeiros-Domingo A, Tester DJ, Vatta M, Farrugia G, Ackerman MJ, Makielski JC. Syntrophin mutation associated with long QT syndrome through activation of the nNOS-SCN5A macromolecular complex. *Proc Natl Acad Sci USA*. 2008;**105**:9355-9360.

Van Goethem G, Luoma P, Rantamäki M, Al Memar A, Kaakkola S, Hackman P, Krahe R, Löfgren A, Martin JJ, De Jonghe P, Suomalainen A, Udd B, Van Broeckhoven C. POLG mutations in neurodegenerative disorders with ataxia but no muscle involvement. *Neurology*. 2004;**63**:1251-1257.

Vatta M, Ackerman MJ, Ye B, Makielski JC, Ughanze EE, Taylor EW, Tester DJ, Balijepalli RC, Foell JD, Li Z, Kamp TJ, Towbin JA. Mutant caveolin-3 induces persistent late sodium current and is associated with long-QT syndrome. *Circulation*. 2006;**114**:2104-2112.

Vatta M, Mohapatra B, Jimenez S, Sanchez X, Faulkner G, Perles Z, Sinagra G, Lin JH, Vu TM, Zhou Q, Bowles KR, Di Lenarda A, Schimmenti L, Fox M, Chrisco MA, Murphy RT, McKenna W, Elliott P, Bowles NE, Chen J, Valle G, Towbin JA. Mutations in Cypher/ZASP in patients with dilated cardiomyopathy and left ventricular non-compaction. *J Am Coll Cardiol*. 2003;**42**:2014-2027.

Vicart P, Caron A, Guicheney P, Li Z, Prévost MC, Faure A, Chateau D, Chapon F, Tomé F, Dupret JM, Paulin D, Fardeau M. A missense mutation in the alphaB-crystallin chaperone gene causes a desmin-related myopathy. *Nat Genet*. 1998;**20**:92-95.

Wang Q, Curran ME, Splawski I, Burn TC, Millholland JM, VanRaay TJ, Shen J, Timothy KW, Vincent GM, de Jager T, Schwartz PJ, Toubin JA, Moss AJ, Atkinson DL, Landes GM, Connors TD, Keating MT. Positional cloning of a novel potassium channel gene: KVLQT1 mutations cause cardiac arrhythmias. *Nat Genet*. 1996;**12**:17-23.

Wang Q, Shen J, Splawski I, Atkinson D, Li Z, Robinson JL, Moss AJ, Towbin JA, Keating MT. SCN5A mutations associated with an inherited cardiac arrhythmia, long QT syndrome. *Cell*. 1995;**80**:805-811.

Watanabe H, Koopmann TT, Le Scouarnec S, Yang T, Ingram CR, Schott JJ, Demolombe S, Probst V, Anselme F, Escande D, Wiesfeld AC, Pfeufer A, Kääb S, Wichmann HE, Hasdemir C, Aizawa Y, Wilde AA, Roden DM, Bezzina CR. Sodium channel β1 subunit mutations associated with Brugada syndrome and cardiac conduction disease in humans. *J Clin Invest*. 2008;**118**:2260-2268.

Watkins H, Conner D, Thierfelder L, Jarcho JA, MacRae C, McKenna WJ, Maron BJ, Seidman JG, Seidman CE. Mutations in the cardiac myosin binding protein-C gene on chromosome 11 cause familial hypertrophic cardiomyopathy. *Nat Genet*. 1995;**11**:434-437.

Xu T, Yang Z, Vatta M, Rampazzo A, Beffagna G, Pilichou K, Scherer SE, Saffitz J, Kravitz J, Zareba W, Danieli GA, Lorenzon A, Nava A, Bauce B, Thiene G, Basso C, Calkins H, Gear K, Marcus F, Towbin JA; Multidisciplinary Study of Right Ventricular Dysplasia Investigators. Compound and digenic heterozygosity contributes to arrhythmogenic right ventricular cardiomyopathy. *J Am Coll Cardiol*. 2010;**55**:587-597.

Yang Y, Xia M, Jin Q, Bendahhou S, Shi J, Chen Y, Liang B, Lin J, Liu Y, Liu B, Zhou Q, Zhang D, Wang R, Ma N, Su X, Niu K, Pei Y, Xu W, Chen Z, Wan H, Cui J, Barhanin J, Chen Y. Identification of a KCNE2 gain-of-function mutation in patients with familial atrial fibrillation. *Am J Hum Genet*. 2004;**75**:899-905.

Yang Y, Yang Y, Liang B, Liu J, Li J, Grunnet M, Olesen SP, Rasmussen HB, Ellinor PT, Gao L, Lin X, Li L, Wang L, Xiao J, Liu Y, Liu Y, Zhang S, Liang D, Peng L, Jespersen T, Chen YH. Identification of a Kir3.4 Mutation in Congenital Long QT Syndrome. *Am J Hum Genet*. 2010;**86**:872-880.
